# *Architector* for high-throughput cross-periodic table 3D complex building

**DOI:** 10.1038/s41467-023-38169-2

**Published:** 2023-05-15

**Authors:** Michael G. Taylor, Daniel J. Burrill, Jan Janssen, Enrique R. Batista, Danny Perez, Ping Yang

**Affiliations:** Theoretical Division, Los Alamos National Laboratory, Los Alamos, NM 87545 USA

**Keywords:** Ligands, Ligands, Cheminformatics, Structure prediction

## Abstract

Rare-earth and actinide complexes are critical for a wealth of clean-energy applications. Three-dimensional (3D) structural generation and prediction for these organometallic systems remains a challenge, limiting opportunities for computational chemical discovery. Here, we introduce *Architector*, a high-throughput in-silico synthesis code for s-, p-, d-, and f-block mononuclear organometallic complexes capable of capturing nearly the full diversity of the known experimental chemical space. Beyond known chemical space, *Architector* performs *in-silico* design of new complexes including any chemically accessible metal-ligand combinations. *Architector* leverages metal-center symmetry, interatomic force fields, and tight binding methods to build many possible 3D conformers from minimal 2D inputs including metal oxidation and spin state. Over a set of more than 6,000 x-ray diffraction (XRD)-determined complexes spanning the periodic table, we demonstrate quantitative agreement between Architector-predicted and experimentally observed structures. Further, we demonstrate out-of-the box conformer generation and energetic rankings of non-minimum energy conformers produced from *Architector*, which are critical for exploring potential energy surfaces and training force fields. Overall, Architector represents a transformative step towards cross-periodic table computational design of metal complex chemistry.

## Abstract (web summary)

Rare-earth and actinide complexes are critical for a wealth of clean-energy applications but Three dimensional (3D) structural generation and prediction for these organometallic systems remains challenging. Here, the authors propose a high-throughput in-silico synthesis code for s-, p-, d-, and f-block mononuclear organometallic complexes.

## Introduction

Data-driven methods for materials^1–3^ and chemical^4,5^ discovery have enjoyed considerable success, fueled by the availability of accurate high-throughput structure-based simulation approaches^6–9^. For example, the dramatic increase in availability of computational data for d-block organometallics was in part enabled by the creation of reliable three-dimensional (3D) structure generation tools; notable examples being the *molSimplify*^6^, *molAssembler*^7^, and *DENOPTIM*^8^ codes. These tools allow for the generation of 3D configurations of organometallic complexes, whose properties can subsequently be computed using high-level electronic structure calculations. *molSimplify* structure generation operates using force-field pre-optimized ligands and assembly of those ligands through alignment to a specified (typically octahedral) metal-center geometry. *molAssembler* operates by leveraging graph enumeration^9^, stereopermuters, and the distance geometry^10^ algorithm informed by tabulated bond lengths. Another approach for organometallic structure generation is taken by the *DENOPTIM*^8^ program, which leverages fragment building in addition to genetic algorithms to assemble hypothetical complexes that optimize a given fitness function. The generation of large numbers of potential complexes, via tools such as the ones listed above, can fuel the computational creation of massive datasets of chemical properties. This, in turn, can aid the development of powerful data-driven machine-learning approaches for the efficient exploration of chemical space.

A complex building algorithm to set up initial structures of lanthanoid complexes has been published by Munguba and co-workers^11^. It considers stereo control, including stereoisomer identification and coordination chirality recognition, and was demonstrated for monodentate and bidentate ligands. f-block organometallic chemistry is vital for clean energy and the nuclear energy cycle, including chemical separations for waste treatment^12,13^, as well as the extraction of rare earth elements^14^ needed for modern technologies ranging from cell phones to turbines^15^. The relative rarity of data on f-block systems can be explained by a range of fundamental and practical factors, including difficulties inherent to working with radioactive elements, elemental scarcity^15,16^, complexity in terms of characterization^17–19^, high coordination environment, and their complex electronic structure^20,21^. Nonetheless, there are noteworthy cases where computational design of organometallics has been successfully performed^22,23^, albeit with structures intuitively-generated by hand and with ligand sets on the order of tens of ligands rather than hundreds or thousands. With these methods, however, large-scale chemistry and structure generation including conformers for potential f-block organometallic complexes remains prohibitively time-consuming, limiting the potential application of large-scale data-driven methods for f-block organometallic chemistries. Because of these challenges, the amount of data on the chemical structure of f-block organometallics is dwarfed by data pertaining to related d-block organometallics^24–26^.

Older 3D-generation packages were benchmarked with root-mean-square displacement (RMSD) comparisons to experimental structures^27^ while recent 3D structure generation routines have not compared generated structures to experimental structures either due to interests more targeted towards specific application spaces^6,8^, or due to concerns surrounding metrics for comparison^7^. Notably, gas phase or liquid phase conformers can dramatically differ from crystalized structures^28^, but different conformers can be vital for accurately capturing spectroscopic^29^ and even catalytic^30^ properties of the underlying complex. Key advances for conformer sampling and the generation and prediction of thousands of structures are the broadly applicable semi-empirical extended tight binding (*xTB*) *xTB*/GFN2-xTB^31^ and related geometry relaxation routines^32^. Both were leveraged to produce the largest available electronic structure-based d-block dataset derived directly from experimental structures^25^. Further methods for enhanced conformer sampling beginning from existing 3D structures show promise for d-block applications^33^. Studies of d-block conformers, generated from a limited set of CSD geometries (~40 structures), revealed lower-energy structures for 68% of CSD structures without any shifts in relative ligand positioning around the metal center^34^. However, with multiple ligands and different symmetries, additional lower-energy solution-phase conformers for even these simpler d-block systems exist, much less with higher-coordinated f-block systems^35^. The availability of large databases of electronic and chemical structures of different conformers and off-equilibrium structures of f-block complexes could drive a more systematic exploration of this vast chemical space. Such a database would also enable the development of powerful accelerated computational methods such as tight binding^36,37^ and machine-learning (ML) potentials^38,39^ capable of capturing f-block long timescale physicochemical properties such as diffusion coefficients and solution-phase reaction rates in f-block extraction processes.

To address the challenges inherent in f-block organometallic structure generation and their relative data paucity, we here introduce *Architector*, a python package for mononuclear organometallic 3D conformer assembly from 2D-inputs based on metal-center symmetry analysis, distance geometry, fragment assembly, and *xTB*-evaluation. To sensibly measure deviations between generated structures where metal-distal configurations overaccentuate differences, we tailor a simple RMSD approach limited to metal-center proximal alignment. Leveraging this RMSD method and energetic comparisons, we show *Architector* can reproduce X-ray diffraction (XRD)-determined 3D chemical structures from the Cambridge Structural Database^26^ (CSD) for f-block in addition to s-, d-, and p- block chemistries. We further show how leveraging tight binding methods inside the Architector generation workflow allows for near-density functional theory (DFT) levels of accuracy in ordering generated conformers out of the box. Finally, we highlight its high-throughput capabilities, producing up to 20 conformers evaluated with *xTB*/GFN2-xTB for each of over 6,000 CSD structures within 12 h on ~500 cores.

## Results

### Architector workflow and design

**Fig. 1:**
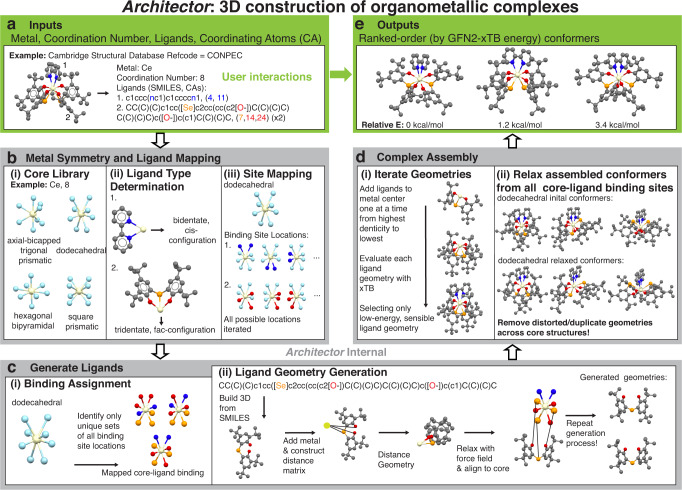
Flow chart of *Architector*: 3D construction of organometallic complexes. **a** Example inputs for an Architector generation derived from a Cambridge Structural Database (CSD) molecule **b** Metal-center symmetry and ligand mapping procedures done in order (i)-(iii), steps (ii) and (iii) are repeated for each core geometry. **c** Ligand binding site assignment mapping (repeated for each core geometry) followed by ligand geometry generation for each binding site mapping. **d** Full complex conformer assembly and evaluation performed for each core geometry and unique binding site mapping. **e** Outputs from the *Architector* generation including multiple conformers sorted by GFN2-xTB energy. Note that users interact directly with only **a** and **e** while **b**, **c**, **d**, and **e**. are handled internally by *Architector*. Inset images are molecules colored by atom type with N:blue, Ce:yellow, C:gray, O:red, Se:orange, and core geometry binding site:light blue, hydrogens have been removed for clarity.

We begin with an overview of *Architector* structure generation along with example chemical illustrations of each step of the process (Fig. 1). We showcase a Ce complex for example inputs (Fig. 1a) and outputs (Fig. 1e) due to its variety of multidentate ligands (both bidentate cis and tridentate fac). The example *Architector* inputs are derived from an existing molecule by identifying the metal, coordination number (CN), ligand simplified molecular-input line-entry system (SMILES) strings^40^, and which index/indices from the SMILES are coordinating atoms (CA) to the metal center. *Architector* includes a utility to aid users in identifying CAs for arbitrary ligand SMILES (Supplementary Fig. 1). In total, *Architector* inputs amount to a full 2D molecular graph specification for the 3D molecule to be constructed.

2D molecular graphs can be defined for any synthesized complex and any not-yet-synthesized complex from across the periodic table for *Architector* structure generation. For user ease in generating 2D molecular graphs and assigning electronic states to complexes, *Architector* also contains default information for each metal including oxidation states, spin, and coordination numbers in addition to a substantial and increasing number (~100) of named ligands that can be used in construction (Supplementary Table 5). After the *Architector* 3D construction, the user is returned a list of generated conformers (Fig. 1e) for the given input, which is ranked according, by default, to their GFN2-xTB^31^ energy.

As deciding if a 2D molecular graph can be embedded into 3D is NP-hard^41^, we opt for heuristic approaches. These approaches attempt to identify close-to-minima energy structures as well as a range of reasonable higher-energy structural isomers that can be useful for understanding high-temperature solution chemistries and for training sets for reduced-cost semi-empirical approaches^37^. For the given metal type and CN, all pre-defined core geometries (Supplementary Table 1) are referenced and tested (Fig. 1b,i). As with the default core library, we identified default ligand types and corresponding CA-M-CA angles (Fig. 1b,ii) from ligands sampled across the CSD (Supplementary Note 1, Supplementary Fig. 2, and Supplementary Table 2). Finally, with the given ligand and coordinating atoms, ligand types can be assigned using an included aid (Supplementary Fig. 3) or from a built-in brute-force search method. Thus, both ligand and core geometries are assigned or determined by *Architector*.

Given the ligand and core geometries, *Architector* constructs all possible mappings between the two. *Architector* determines all ligand binding sites by taking all combinations of core CA positions and determining if the angles between all pairs of CAs align with the assigned ligand geometry (Fig. 1b,iii and Supplementary Fig. 4). Then, *Architector* assigns valid sets of binding sites from all possible binding sites for all ligands by reducing to only sets of possible binding sites with no shared core CAs (Fig. 1c,i).

Even for simple complexes, the number of possible core-ligand binding site mappings can be enormously large, needing that one only tests a reduced number of mappings. For example, a CN = 8 complex bound by 8 different monodentate ligands has 8! = 40,320 possible core-ligand binding site maps. However, if some of the ligands are identical (e.g., 4 water and 4 ethanol ligands), the number of unique mappings usually decreases by 2–3 orders of magnitude (8 choose 4 = 70). Thus, for cases with fewer distinct ligands, mappings can automatically be greatly reduced.

Mappings can be further reduced by not considering structures with identical metal-center symmetries. To evaluate the uniqueness of valid sets of binding sites and quickly rank the likelihood of each mapped core-ligand binding, we developed a heuristic function corresponding to a pseudo-energy of binding sites (Supplementary Note 2). We note that the pseudo-energy evaluated with *b* = 1 heuristic charge produced ~94% of GFN2-xTB lowest-energy conformers considering only the top 10 lowest-pseudo-energy binding configurations (Supplementary Fig. 5), while using up to the top 19 lowest-pseudo-energy configurations produced 99.5% of lowest-energy conformers. Therefore, pre-calculating pseudo-energy greatly reduces the number of sets of binding sites that must be considered if only minimum-energy structures are desired.

*Architector* 3D generation of structures then starts from ligands and assigned ligand CA binding sites (Fig. 1c,ii). We developed the ligand 3D-generation routines extending and synergizing approaches from both *molSimplify*^6^ and *molAssembler*^7^. First, we initialize chemically-meaningful ligand geometries from the SMILES strings using the *Openbabel*^42^ package, which are then relaxed with either MMFF94^43^ or UFF^44^. Next, we prepare ligands for distance geometry^45^ conformer generation^46^. Briefly, we form both lower-bound and upper bound distances based on the initial ligand geometries in addition to single bond covalent radii (*r*_cov_)^47^, and van der waals radii^48^ (vdw) found in the literature. Additional details surrounding distance matrix construction and distance geometry generation can be found in Supplementary Table 3 and Supplementary Note 3. To correct the distortions resulting from distance geometry, we turn again to FFs with a multistep geometry cleaning process (Supplementary Note 4). Following FF cleaning, the Kabsch algorithm^49^ is used to rotate the ligand around the metal to minimize CA location deviation from the assigned core geometry locations, returning the rotational root-mean-squared deviation (rotRMSD) between the sets of locations (Supplementary Note 5). After force-field relaxation and alignment, the generated ligand conformers are ready for metal-ligand complex assembly.

With different ligand conformations at multiple sites at the metal surface, rapid methods for ranking conformations and reducing numbers of structures breaking chemical sanity are needed for complex assembly. By default we use GFN2-xTB^31^ to select FF-relaxed ligand geometries by placing each ligand conformer on the complex and evaluating the total energy to select low-total-energy geometries for each ligand in order from highest to lowest denticity (Fig. 1d,i). To bias the conformers to their assigned binding sites, we select the conformers with the lowest total GFN2-xTB energy multiplied by 1/rotRMSD to minimize potential ligand overlaps. As this bias does not remove all potential ligand overlaps, we introduce three distinct interatomic distance cutoff checks before GFN2-xTB evaluation to minimize tests on unreasonable geometries (Supplementary Note 6, Supplementary Fig. 6, and Supplementary Table 4). If different methods for ordering of ligands during construction are desired, users can optionally request all the assembled complexes and specify any other desired ordering (Supplementary Fig. 7). Assembled complexes are then ready for relaxation and electronic structure analysis.

Final relaxation on assembled complexes can be performed with any electronic structure or force-field method, though by default *Architector* uses GFN2-xTB. If unspecified by the user, *Architector* assigns default oxidation state, spin state, and core CNs (Supplementary Table 5) to the complex based on the metal identity and total electron counts (Supplementary Note 7). For the actinides, we swap the metal center and electron counts for the equivalent-group lanthanide, since atomic numbers greater than 88 are not supported by GFN2-xTB^31^. However, this swap is unnecessary for models parametrized for actinides^37^. With charge and spin assigned, we relax all assembled complexes within the Atomic Simulation Environment (*ASE*)^50^. Once relaxed, we perform an additional set of tighter interatomic distance chemical sanity checks ensuring generated structures match the input 2D molecular graph (Supplementary Table 4). For all relaxed complexes found to satisfy the input parameters, *Architector* by default applies Kuhn–Munkres ordered RMSD (kmRMSD)^51^ to eliminate duplicate structures before returning all generated complexes ranked by GFN2-xTB energy (Fig. 1e). Additional comments surrounding assumptions made during complex construction can be found in Supplementary Note 8. From input to output structures, *Architector* is thus uniquely tailored to high-throughput and accurate organometallic complex 3D structure generation.

### Structure reference for targeted generation and CSD-replication set

**Fig. 2:**
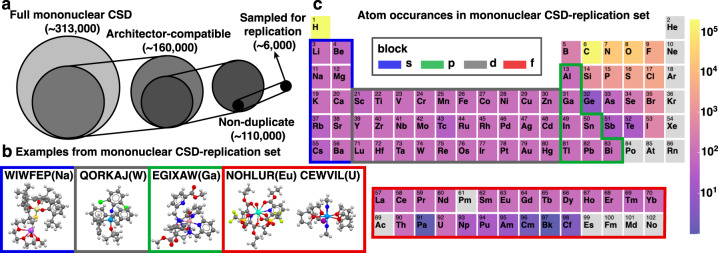
Cross-periodic table replication dataset construction. **a** Size of different subsets of the CSD resulting in the sample set for replication. **b** Examples structures of sampled replication set with Cambridge structural database (CSD) refcode(metal) indicated. **c** Atom distributions present across all structures sampled from the CSD divided by blocks. Inset images are molecules colored by atom type with Na:light purple, F/Cl:green, I:dark purple, N:blue, C:gray, Si:light yellow, O:red, S:yellow, H:white, Ga:brown, W/Eu/U:light blue.

To create a targeted set of mononuclear complexes for 3D structure generation, we turned to the CSD^26^. We mined the CSD version 5.43 (Nov 2021) for mononuclear structures (Fig. 2a and Supplementary Note 8) using previously described methods^52^, resulting in a total of 312,527 mononuclear complexes. The full CSD distributions of mononuclear metal centers are 83% d-block with 80% CN less than 8 and primarily coordinated by C, N, or O atoms (Supplementary Fig. 8). From here, the *CSD python API* was used to remove structures incompatible with *Architector*, broadly due to mismatches in stoichiometry between the 2D representation and 3D structures, yet-unsupported ligands, unreasonable charges, or structures with distorted geometries (Supplementary Table 6). We highlight example *Architector*-compatible structures from CSD (Fig. 2b) that are representative of different blocks, visually noting that f-block complexes tend to be higher-coordinated. To focus on a more evenly distributed set of complexes across metal (some metals have fewer than ten structures), we randomly sampled up to 100 different complexes for each metal resulting in a subset of 6,154 structures for generation (Fig. 2c and Supplementary Table 7). The atom distributions and descriptive statistics for this subsampled set are still largely representative of the full CSD (Supplementary Fig. 8 and Supplementary Fig. 9). Thus, we created a cross-periodic table set of organometallic complexes for replication by *Architector*.

### Structure and energy statistics for lowest-energy *Architector* conformers vs. CSD

Across the full set of 6,154 structures, we performed *Architector* generation using CSD-derived oxidation state assignments (Supplementary Note 7) and corresponding spin states suggested by the *mendeleev*^53^ package and relaxed the CSD-derived structures with GFN2-xTB. Overall, this resulted in 5,956 successful generation of sane relaxed complexes with comparable successfully-relaxed GFN2-xTB CSD structures (Supplementary Fig. 10 and Supplementary Table 8). Accounting for unsuccessful generations that do not result directly from *Architector* failures (Supplementary Note 9), only 57 structures were unable to be handled by *Architector* construction, corresponding to a 99.1% success rate in generation. Comparable statistics on organometallic structure generation for the *CORINA* program^54^ was 51.6% successful generation, while the *COSMOS* program reported comparable 94.6%, the only other programs benchmarked in a similar manner^27^. Importantly, this prior benchmark organometallic set^27^ did not include any structures with CN > 8, where actinides and lanthanides constitute the majority of observed complexes (Supplementary Fig. 8). Thus, *Architector* is directly competitive with state-of-the-art comparable programs even on complex actinide and lanthanide structures.

**Fig. 3:**
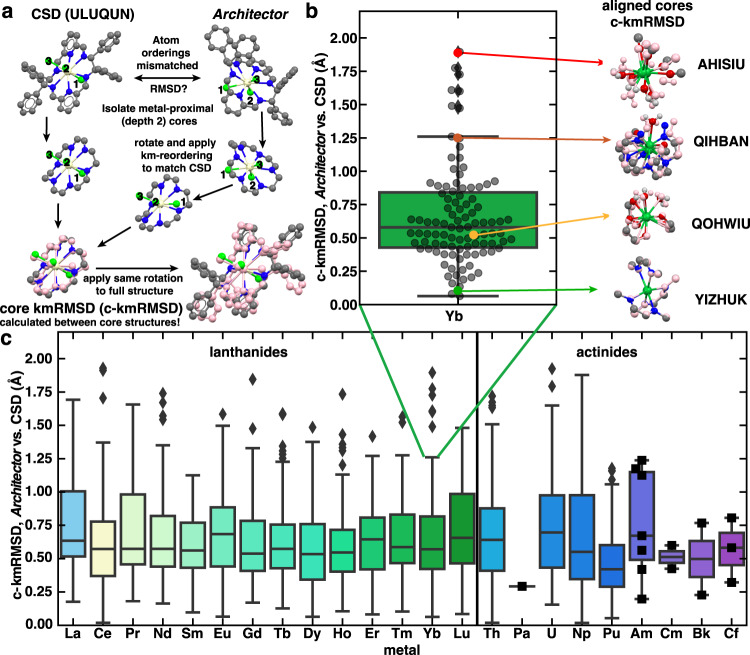
Replication of f-block CSD structures. **a** Illustration of core Kuhn–Munkres root-mean-squared distance (c-kmRMSD) alignment procedure **b** Single boxplot distribution of minimum-c-kmRMSD Architector-generated structures vs. CSD c-kmRMSD values for Ytterbium complexes. C-kmRMSD values are calculated only for the atoms within depth 2 neighbors of the metal center. Insets highlight closely mapped, average, and less-ideally mapped generated cores. **c** Boxplot distributions of c-kmRMSD of all minimum-c-kmRMSD Architector-generated lanthanide and actinide complexes. All insets the CSD structures are colored by atom type with Yb:green, Cl:light green, N:blue, C:gray, O:red, Ce:yellow, H:white. Overlayed mimimum-*c-*kmRMSD Architector-generated structures are colored pink to reveal differences in structure. Boxplots contain a colored region indicating the inner quartile range (IQR) between the 1st and 3rd quartiles, while whiskers indicate points within 1.5*(IQR) of either the 25 and 75 percentile points, and black diamonds indicate outliers beyond the 1.5*IQR cutoffs. Metals with fewer than 10 structures show all datapoints in black squares.

Focusing further on the f-block structures generated, we need reliable and descriptive methods for describing how close the generated structures are to the CSD reference structures. A tricky aspect of comparing different organometallic structures in terms of energy and structures is that variations in soft trailing organic components of the molecules can obscure a direct RMSD comparison between two organometallics, leading to identical graphs and visually/chemically similar structures being artificially labeled as distinct^7^. To directly compare the structures, we leverage the kmRMSD method specifically in the region proximal to the metal center within two graph hops of the metal center on the molecular graph (Fig. 3a). The kmRMSD up to depth two from the metal center was selected (c-kmRMSD), as this depth shows a stronger correlation with energy differences (Supplementary Fig. 11).

To illustrate how c-kmRMSD depicts differences in structures and how it trends with chemical intuition, we focus on Yb-generated compounds showing a wide spread in c-kmRMSD. For example, the *Architector*-generated Yb structure with c-kmRMSD near 2.00 Å has a metal-center symmetry with different ligand relative locations (Fig. 3b). Decreasing c-kmRMSD to near 1.25 Å reveals a non-distorted Yb structure with only one different relative ligand position than the CSD-derived structure. Looking at a structure near the mean c-kmRMSD (0.5 Å for Yb) reveals a close-to-perfect match in structure with only deviating hydrogen positions. Finally, looking at an example Yb structure with c-kmRMSD near 0.1 Å reveals virtually identical core and full structures. Broadly, this agrees with previous reports of RMSD similarity where RMSD < 1 Å can be regarded as identical conformers^27^. Vitally, higher c-kmRMSD often does not imply chemically unreasonable structures, merely differing ligand symmetries. Utilizing c-kmRMSD thus allows us to compare metal-centered structural comparison between generated and CSD-reference structures.

With c-kmRMSD, we primarily focused on the lanthanide/actinide f-block targets looking to generated conformers closest to the CSD equivalents (Fig. 3c). We immediately note that across the lanthanides and actinides, minimum c-kmRMSD values average near 0.5 Å indicating *Architector* typically generates at least one structure with minimal deviations between the generated structures and CSD structures. Lanthanides and actinides missing from Fig. 3c are not found at all in the CSD (Supplementary Fig. 8). We see that though actinides were simulated in GFN2-xTB by replacing them with their f-electron equivalent lanthanides, the average c-kmRMSDs of actinides are comparable to those across the lanthanides. Additionally, only 35 of 1,645 f-block structures fall in the >1.5 Å c-kmRMSD region where the matching ligand symmetry to the CSD was not generated via Architector. We further note that across the whole periodic table there is similar average and worst-case c-kmRMSD values between Architector-generated and CSD-derived structures (Supplementary Fig. 12). Overall, these results highlight a great degree of agreement between the most similar Architector-generated complexes to CSD-derived structures they are replicating.

**Fig. 4:**
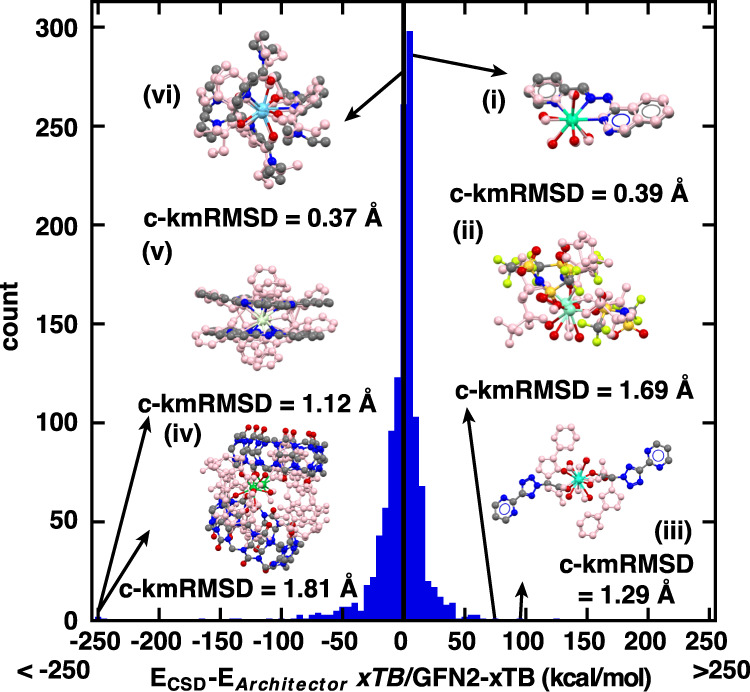
Lanthanide CSD vs. *Architector* GFN2-xTB energetics. Histogram of the difference in energies of the GFN2-xTB-relaxed CSD structure and the lowest-energy Architector conformers on lanthanide complex energetics. (i)–(vi) highlight examples of structures falling into extreme categories. (i) and (vi) represent Architector structures nearly isoenergetic with the CSD structure while (ii) and (iii) are > 50 kcal/mol more stable than the CSD structure and (iv) and (v) are > 250 kcal/mol less stable than the CSD structure. All insets the CSD structures are colored by atom type with Lns:green/light blue, F:light green, N:blue, C:gray, O:red, S:yellow. Overlayed minimum-*xTB*-energy *Architector*-generated structures are colored pink to reveal differences in structure with hydrogens removed for clarity. Associated c-kmRMSD values on the insets provide a measure of metal-centered similarity between the structures.

Beyond structural comparisons, energetic comparisons via GFN2-xTB can also be made between the Architector-generated structures and those from the CSD. With trailing-ligand conformations potentially playing a larger role in determining energetics than metal-proximal structure, we expect deviations in energetics between minimum-energy Architector-generated structures vs. CSD structures. We find instead that the mean absolute *xTB* energy difference on the minimum-energy Architector structures and the CSD structures is 12.1 kcal/mol (Fig. 4) with 96.1% of structure energies within 50 kcal/mol of the CSD structure. Additionally, the average difference between the CSD and Architector structures is −4.6 kcal/mol, indicating the Architector structures are, on average, only slightly higher in GFN2-xTB energy than the CSD structures. The trends and histogram of lanthanide energetics is also closely mapped by s-, p-, and d- block Architector-generated complexes as well (Supplementary Fig. 13). Overall, the *xTB* energetics of minimum-energy Architector structures are comparable with CSD structure energetics.

Instead of overall energetic trends, focusing on specific cases that both match and deviate in energy reveals potential sources of larger energetic discrepancies between Architector and the CSD. Architector structures much lower in energy (>50 kcal/mol lower) than the CSD structures seem to have bulkier ligands for which Architector identifies different ligand orientations that are lower in energy (Fig. 4ii/iii). These structures show c- kmRMSD values >1 Å indicating the core geometries are nearly identical, but ligand conformations differ, indicating crystal packing likely influenced the CSD conformer structure^55^. Alternatively, structures for which the lowest-energy Architector structure was much higher in energy (>250 kcal/mol higher) than the CSD structures seem to be cases where Architector-generated a distorted ligand conformation different from that observed in the CSD (Fig. 4iv/v), albeit close core structures. However, only 33 out of 344 lanthanide structures with more than 100 atoms show >50 kcal/mol higher energy than the CSD structure. The structures much higher in energy than the CSD tend to have larger ligands, which have more rotational/structural degrees of freedom.

The *CREST* utility represents a powerful conformer sampling utility also applicable to lanthanide complexes targeted to sample local rotational/structural degrees of freedom. Comparison of *CREST* sampling performed directly on 19 CSD structures to Architector-generated structures revealed *CREST* generating structures lower in energy than the CSD structure in 83% of the cases with energy distributions on the order of 10 kcal/mol (Supplementary Note 11 and Supplementary Fig. 14). Since Architector represents a structure search over more diverse configurations, it produced structures lower in energy than the CSD in 84% of cases, with energy distributions on the order of 20 kcal/mol. The combination of these utilities, with *CREST* sampling performed after Architector generation is already implemented in Architector and is very likely to produce structures at or lower in energy than the CSD for a given molecule, though it typically adds between 10–15 min of run time per complex to the generation procedure. We note that even without any additional conformational sampling via CREST, Architector identifies a majority (55.1%) of conformers for f-block lanthanides within typical *xTB* accuracy (2 kcal/mol)^31^ or lower in energy than the CSD structure. Additionally, although outliers in energetics exist, 64.5% of lowest-energy lanthanide *Architector* structures are within 10 kcal/mol (nearly isoenergetic and isostructural) with the CSD structures (Fig. 4i/vi). Finally, CREST requires a user-estimated 3D input structure for a molecule to perform conformer sampling, while *Architector* only requires 2D graph information about the molecule as input and thus can be easily applied to any metal-ligand combinations.

### Larger Architector conformer space

Besides lowest-energy conformers, Architector can generate ranked-order conformers by GFN2-xTB energy. Such higher-energy conformers can be vital for understanding and accelerating f-block complex design. From a randomly-selected set of 765 complexes from across the periodic table we isolated 3,039 conformers generated by Architector to test with DFT relaxation methods (Methods Section and Supplementary Fig. 15). We note that since GFN2-xTB does not support atoms with *Z* > 88, an approximation for the actinides is needed for comparison of GFN2-xTB conformer energetics and structures. In Architector and for comparison to the CSD, actinides are approximated by swapping the actinide with the same-group lanthanides.

**Fig. 5:**
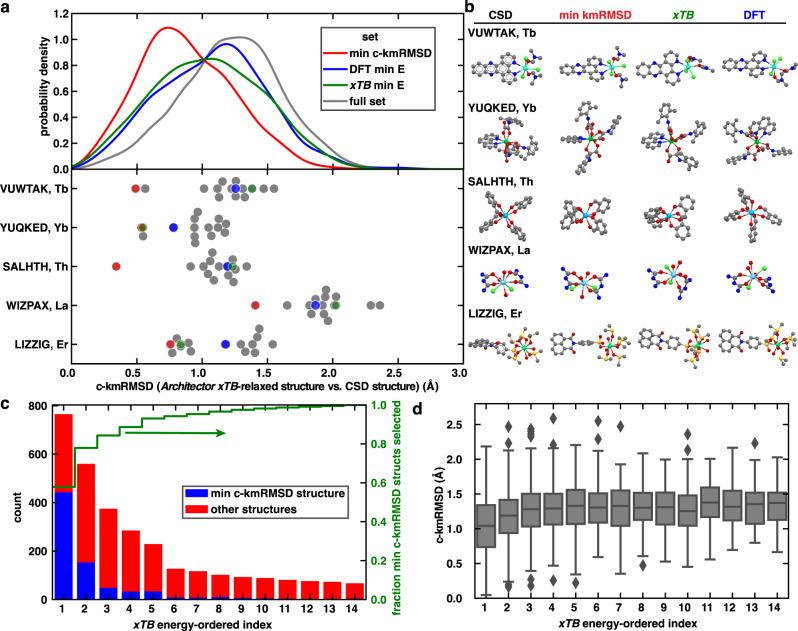
Conformer distributions and examples. **a** Kernel density estimate (KDE) plot of distributions of *Architector*-generated un-relaxed conformer core kmRMSD vs. CSD structures over the full set of 3039 structures from 765 different complexes (upper). (lower) Specific examples of structures where more than 13 conformers were relaxed by both DFT (PBE + D/DZP) and *xTB* (GFN2-xTB) and the distribution of their core kmRMSD values. **b** Renderings of the conformations corresponding to the structure directly from the CSD under the CSD refcode, the closest *Architector*-generated un-relaxed initial conformer, the minimum-energy *xTB*-relaxed conformer, and the minimum-energy DFT-relaxed conformer. Calculations for actinides in *xTB* are reported with a lanthanide used as proxy. All renderings are colored by atom type with Lns:green/light blue, Cl:light green, N:blue, C:gray, O:red, S:yellow. **c** *xTB* energy-ordered index vs. fraction of minimum c-kmRMSD conformers and **d** *xTB* energy-ordered index vs. c-kmRMSD for all conformers evaluated with both *xTB*/DFT.

Crucially, in this work we are not intending to focus on accuracy of post-Architector methods or even the underlying *xTB* methods. From the electronic structure standpoint, we instead are focused on whether the Architector-generated structures are able to be evaluated by general electronic structure methods, and whether, from a given method these structures are energetically and geometrically sensible compared to experimentally-derived structures. For more detailed studies of specific regions of the periodic table any available method can be utilized to evaluate the configurations following Architector generation ranging from force fields (UFF) to complete active space self-consistent field (CASSCF). Users can readily customize both Architector internal methods (e.g., ASE Calculator (ADF/Gaussian/VASP), functional, and/or basis set) used and final evaluation methods to their own purposes. Given this broader goal and a desire to minimize computational expense, we have chosen to report PBE^56^ + D^57,58^/DZP methods^59^ as the baseline *xTB* vs. DFT comparison (Fig. 5).

Over *Architector* structures, we were able to perform single point calculations on as many as 97% of all conformers with DFT including 94% of lanthanide and 99% of actinide conformers attempted (Supplementary Fig. 15). Looking instead at distinct complexes, we were able to perform at least one successful DFT relaxation from 99% of lanthanide complexes and 100% of actinide complexes. Lanthanide complexes are known to be particularly difficult to converge in DFT^60–62^, so a lower rate of success is to be expected and is consistent with convergence on CSD structures themselves (Supplementary Fig. 15). Thus, for most f-block complexes, and especially actinide complexes (even considering the approximation of swapping for lanthanides in GFN-xTB evaluation), *Architector* is generating structures amenable to DFT minimization.

Beyond the minimum-energy PES structures, we benchmarked *Architector* and the underlying GFN2-xTB ability to rank conformers in terms of energy and structure (Fig. 5). Over the full set of complexes and conformers evaluated with DFT (3039 conformers), we see that often the closest c-kmRMSD structure (vs. CSD) is not the lowest-energy for either DFT or GFN2-xTB (Fig. 5a). This again supports previous observations that minimized gas and liquid phase complex conformations do not necessarily correspond to those observed in single-crystal structures^28^. These observations suggest that the distribution of conformers generated by *Architector* is key for capturing configurations important for different theoretical methods.

Looking at specific examples where more than thirteen conformers were able to be relaxed also provides insight into factors dictating minimum-energy structures for each method (Fig. 5b). Here, we directly see that *Architector* is typically generating at least one conformer quite close in structure to the CSD, but also forming a wide distribution of conformers in terms of metal-core local geometry (Fig. 5a). Both GFN2-xTB and DFT favor structures with two key differences from the CSD. First, for structures DFT/GFN2-xTB VUWTAK, SALHTH, LIZZIG, and WIZPAX, different ligand mappings and metal-center symmetries are observed from the CSD structure (Fig. 5b). This type of deviation potentially agrees with previous catalytic work where the catalytically active and lowest-energy structures had different relative ligand symmetries^63^. We note that accessing these types of conformers directly from an XRD initial structure is difficult, if not impossible^34^. The second type of deviation is primarily observed in the YUQQED structures, where the metal-ligand symmetry is nearly the same as the experimental structure, but trailing-ligand orientation differs resulting in a lower-energy conformer. Additionally, we note that oxidation states for lanthanides and actinides differing from the +3 state were also sampled and *Architector*-produced structures for these compounds were comparable to experimental structures (e.g., SALHTH in Fig. 5a/b). Since nearly all the multi-symmetry (more than 13 conformers) complexes are from the f-block, ligand symmetry effects appear especially important when considering actinide and lanthanide complexes as enabled in *Architector*.

Another key question relevant to structural generation is how many conformers are needed to produce structures closest to experimental structures. Here, we note that *xTB* energy-index ordering tends to trend strongly with minimum c-kmRMSD structures with over 93.3% of minimum c-kmRMSD structures being found in the top 5 lowest-*xTB*-energy conformers (Fig. 5c). Additionally, we directly observe that *xTB* energy-ordered index also trends directly with c-kmRMSD with lower indices tending to have lower c-kmRMSD values (Fig. 5d). Beyond closeness to experiments, *xTB*-ordered index also appears to trend with DFT-evaluated relative energy (Supplementary Fig. 16). Together, these observations indicate that the combination of *Architector/xTB* tends to select for structures that are close to experiments but potentially contain some key differences in ligand symmetry, resulting in the sampling of lower-energy structures.

### High-throughput capabilities

**Fig. 6:**
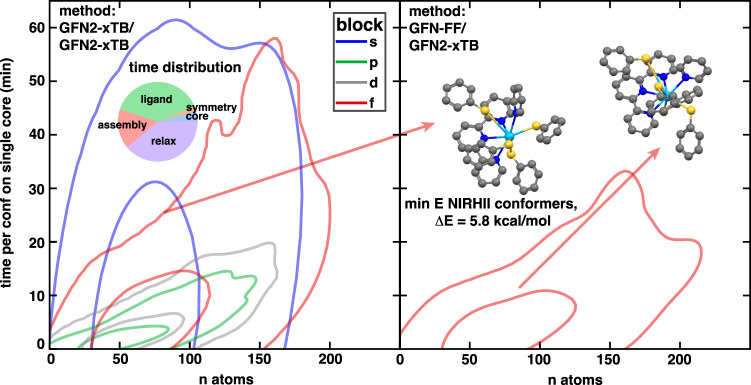
Timings and Performance of *Architector*. 2D Kernel Density Estimates (KDE) of the time per configuration on a single conformer on a single core vs. number of atoms (n atoms) by each block. The smaller circles represent density estimates of 50% of the data, while the outer circles represent 95% density estimates. The inset on the left indicates the average distribution of the times for each subtask of *Architector* where “core” refers to core and core-ligand mapping determination time, “symmetry” to finding valid sets of core-ligand bindings, “ligand” to ligand conformer generation time, “assembly” to time placing and evaluating ligand conformers, and “relax” to the final relaxation time with GFN2-xTB. The right plot is the same generations performed with GFN-FF for assembly rather than GFN2-xTB for all Ce, Nd, Eu, Ho, Th, and U clusters. The inset highlights the structural results for both methods are nearly identical and are colored by atom type with Th:light blue, N:blue, C:gray, S:yellow.

Assessing the properties of yet-unsynthesized complexes requires rapid screening of large chemical spaces and numbers of conformers. From the set replicating the CSD, we report an average generation time of 6.7 min per valid conformer returned on a single core (Fig. 6 and Supplementary Fig. 17). For structures containing fewer than 100 atoms (~80% of structures), an average of 3.3 min per valid conformer on a single core is achieved. We note that the assembly and relaxation steps of the final complexes dominate the time spent generating complexes. Instead of using GFN2-xTB for both steps, a user can request the force field, e.g., GFN-FF^64^ for the assembly and GFN2-xTB for relaxation, as leveraged in *CREST*^33^. Over the same set of f-block complexes, simply swapping GFN2-xTB for GFN-FF during assembly resulted in a reduction in generation time by a factor of 2 with minimal tradeoff in the quality of the results (Fig. 6).

*Architector* can also be readily run in an “embarrassingly” parallel fashion over complexes, which enabled over 99% of the 6154 structural generations relaxed at the GFN2-xTB level to complete within 12 h on ~500 cores. Given the rapidity of other methods of 2D to 3D-generation such as *CORINA*, which reports around 0.009 min per conformer on a single core over *Pubchem*, this may still seem comparatively slow^54,65^. However, given the complexity of the targeted systems (f-block) along with accounting for spin, charge, and energetics of multiple conformers (not performed with any other package), *Architector* is a very effective tool for high-throughput 2D to 3D generation for challenging complex organometallics across the periodic table.

## Discussion

*Architector* represents a transformative tool for 3D structure generation of mononuclear organometallic compounds, particularly f-block complexes. We have shown it capable of producing chemically valid 3D structures from 2D inputs, producing numerous GFN2-xTB-relaxed conformers for each given input. The energetics and introduced core kmRMSD comparison between the CSD and *Architector*-generated f-block complexes reveal quite similar structures. Due to conformational sampling and crystal field effects, in a majority of cases *Architector* was able to find comparable-energy gas-phase structures to those obtained from the CSD and evaluated by both DFT and GFN2-xTB. With many chemically-sensible conformers generated for each structure and energetic rankings of non-minima energy conformers, *Architector* is ready for use in tasks such as force field and tight binding training for chemical systems where cheaper methods do not yet exist^31,66^. Finally, *Architector* shows strong promise for high-throughput structure generation of mononuclear complexes across the whole periodic table, which can enable the data-driven discovery of complex chemical systems essential for energy, catalysis, separation that are on the horizon.

Although *Architector* is achieving over 97% success in structural generation across the periodic table, as with any 3D-generation routine, *Architector* relies on heuristics and thus is not guaranteed to find the lowest-energy conformer for a given molecule. Further developments in *Architector* are underway to expand its capabilities to include ligand type handling for structures with neighbors bound to the metal center and haptic ligands with CAs not involved in the haptic interaction, representing ~7% of ligands found in the CSD (Supplementary Table 6). In this first version of *Architector*, we focus on mononuclear metal compounds, while extensions to polynuclear structures by linking mononuclear centers are also under development.

Overall, the structural generation and conformer ranking ability of *Architector* is remarkably broad. With conformers readily evaluated with any electronic structure method, the screening of metal spin, metal charge, ligand coordinating geometries, and ligand packing become available, enabling accurate evaluation of properties such as ligand binding energies, spectroscopic properties, and potentially catalytic properties of organometallics. Although primarily developed for higher-coordinated lanthanide and actinide chemistries, *Architector* has proven applicable across the periodic table, dramatically expanding its potential usage. Additionally, though benchmarked against known crystal structures, there is no limitation of *Architector* to known chemical structures as it is readily applied to any metal-ligand complex. Thus, *Architector* represents a vital step towards data-driven discovery in f-block chemistries and beyond.

## Methods

For CSD replication calculations, up to 20 different core symmetries per core-ligand mapping were requested, with the top 10 lowest-energy conformers relaxed by *xTB*/GFN2-xTB. All assembly steps were performed with *xTB*/GFN2-xTB as well except where otherwise indicated. Single point GFN2-xTB energetic evaluations with the methanol (*ε* = 33)^67^ solvent revealed shifts in conformer energetics and energetic rankings (Supplementary Figs. 18 and  19). Shifts are expected given more polar conformers would be more stabilized by the presence of solvents and are suggested for use in cases where solvents are known. In *Architector*, adding solvents supported by *xTB* during complex construction and conformer ranking is facilitated by adding a single “solvent” keyword. For this work, since the structures evaluated are derived from crystals made in different solvents (e.g., *ε* = 2 for hexane to *ε* = 80 for water)^67^, simulating in the gas-phase serves as a common reference. Ligand types were assigned for replication by choosing the ligand type classified from the 3D CSD structure (Supplementary Table 2). Duplicates with full kmRMSD values lower than 0.5 Å were removed. *Architector* relies heavily on several open-source packages including *ASE*^50^, *scipy*^68^, and *xTB*^66^. Internal visualization is supported using the *py3Dmol*^69^ plugin for jupyter notebooks. All molecular renderings were made using the *Mercury*^70^ package from the CCDC.

All DFT relaxations were performed with the *ADF* 2022.101^71^ using the PBE exchange-correlation density functional^56^ managed by the open-source pyiron package^72^. For structures with all elements *Z* < 86, D4 dispersion corrections (+D)^57^ were added, while for structures with all elements *Z* < 95, D3 corrections^58^ were added to facilitate more similar comparison between DFT and *xTB* methods. A smaller double-zeta basis set with polarization functions (DZP)^59^ was used to accelerate DFT calculations and encourage convergence, while ZORA scalar relativistic effects^73^ were employed to capture relativistic effects typically present in f-block elements. Single point comparisons revealed minimal impact of basis set and functional selection on conformer ranking energetics (Supplementary Note 12 and Fig. 20). We evaluated spin contamination on the PBE + D/DZP DFT calculations and found only 7 of 3039 structures contained <*S*^2^ > deviations greater than 0.5 (Supplementary Fig. 21).

### Supplementary information

Supplementary Information

### Source data

Source Data

## Data Availability

All data generated in this study have been deposited in the Zenodo database under accession code 10.5281/zenodo.7764697. The data contained in the figures in this study are provided in the Source Data file. Source data are provided with this paper.

## Code availability

The *Architector* code is available on github at and is also able to be installed via the Anaconda (conda install -c conda-forge *architector*) or pip (pip install *architector*) package managers. A permanent version associated with this manuscript can be found at 10.5281/zenodo.7754279.
