# Author Correction: *Architector* for high-throughput cross-periodic table 3D complex building

**DOI:** 10.1038/s41467-023-42034-7

**Published:** 2023-10-04

**Authors:** Michael G. Taylor, Daniel J. Burrill, Jan Janssen, Enrique R. Batista, Danny Perez, Ping Yang

**Affiliations:** Theoretical Division, Los Alamos National Laboratory, Los Alamos, NM 87545 USA

**Keywords:** Ligands, Ligands, Cheminformatics, Structure prediction

Correction to: *Nature Communications* 10.1038/s41467-023-38169-2, published online 15 May 2023

The introduction of this article states “Up to now, applications of such automated 3D-generation approaches to rare-earth and actinide (f-block) organometallic chemistry have not been reported”. The authors would like to rectify this statement as “A complex building algorithm to set up initial structures of lanthanoid complexes has been published by Munguba and co-workers^11^. It considers stereo control, including stereoisomer identification and coordination chirality recognition, and was demonstrated for monodentate and bidentate ligands.”

[11] Munguba, G. H. L. et al. The complex build algorithm to set up starting structures of lanthanoid complexes with stereochemical control for molecular modeling. *Sci. Rep.* **11**, 21493 (2021).

The original article has been corrected.

