# Supplementary material for: Architector for high-throughput cross-periodic table 3D complex building
Source: Nat Commun. 2023 May 15;14:2786. doi: 10.1038/s41467-023-38169-2 (PMC10185541; doi:10.1038/s41467-023-38169-2)
Supplement: Supplementary file 1 — Supplementary Information [file 41467_2023_38169_MOESM1_ESM.pdf]

Supplementary Information:
***Architector* for high-throughput cross-periodic table 3D complex building**

Authors: Michael G. Taylor[a,#], Daniel J. Burrill[a,#], Jan Janssen[a], Enrique Batista[a,*], Danny Perez[a,*], and Ping Yang[a,*].
[a]Theoretical Division, Los Alamos National Laboratory, Los Alamos, New Mexico, 87545, United States
[#] authors contributed equally.
[*] pyang@lanl.gov, danny_perez@lanl.gov, erb@lanl.gov

**Supplementary Figure 1** *Architector* coordinating atom (CA) determining utility	Page 2
**Supplementary Table 1** Default core geometries	Page 3
**Supplementary Note 1** Ligand classification characteristics	Page 4
**Supplementary Figure 2** Ligand classification procedure	Page 5
**Supplementary Table 2** Ligand type interatomic angles	Page 6
**Supplementary Figure 3** Example ligand type visualization	Page 7
**Supplementary Figure 4** Illustration of ligand-core mapping	Page 8
**Supplementary Note 2** Pseudo-energy definition	Page 8
**Supplementary Figure 5** Histogram of pseudo-energy index vs. *xTB* index	Page 9
**Supplementary Table 3** Distance matrix construction elements	Page 10
**Supplementary Note 3** Distance geometry methods description	Page 10
**Supplementary Note 4** Forcefield cleaning method and CA reassignment	Page 11
**Supplementary Note 5** Kabsch algorithm alignment of ligands	Page 11
**Supplementary Note 6** Interatomic distance sanity checks	Page 11
**Supplementary Figure 6** Illustration of sanity check failures	Page 12
**Supplementary Table 4** Default sanity check cutoffs	Page 13
**Supplementary Figure 7** Illustration of ligand conformer-core mapping	Page 13
**Supplementary Table 5** Default metal oxidation, spin states, and coordination numbers	Page 14
**Supplementary Note 7** How spin and charge states are assigned	Page 15
**Supplementary Note 8** Additional comments on complex construction	Page 15
**Supplementary Note 9** Mononuclear structure mining procedure	Page 16
**Supplementary Figure 8** Distributions of mononuclear complexes in full CSD.	Page 17
**Supplementary Table 6** Filtered complex counts and reasons	Page 18
**Supplementary Figure 9** Distributions of complexes in *Architector*-replication set	Page 18
**Supplementary Table 7** *Architector* replication set oxidation state and spin state counts	Page 19
**Supplementary Figure 10** Cross-periodic table generation success and failure counts	Page 20
**Supplementary Table 8** Sources of failure in generation tabulation	Page 21
**Supplementary Note 10** Description of sources of failure	Page 22
**Supplementary Figure 11** Core depth in kmRMSD correlations	Page 22

Supplementary Figure 12 Core kmRMSD distributions over full replication set Page 23
Supplementary Figure 13 Histogram of generated energies vs. CSD energies Page 23
Supplementary Note 11 *CREST* method parameters. Page 24
Supplementary Figure 14 Comparison to *CREST* Page 25
Supplementary Figure 15 Barplots of DFT successes and failures Page 26
Supplementary Figure 16 Distributions of DFT vs. *xTB* over all DFT-relaxed structures Page 27
Supplementary Figure 17 Distribution of *Architector* time per complex on all complex Page 27
Supplementary Figure 18 Histogram of solvated generated energies vs. CSD energies Page 28
Supplementary Figure 19 Solvated energy index differences Page 28
Supplementary Note 12 Comment on single point energies Page 29
Supplementary Figure 20 Single point energy parity plots for basis set and functional Page 29
Supplementary Figure 21 Spin contamination plot Page 30
Supplementary References Page 30

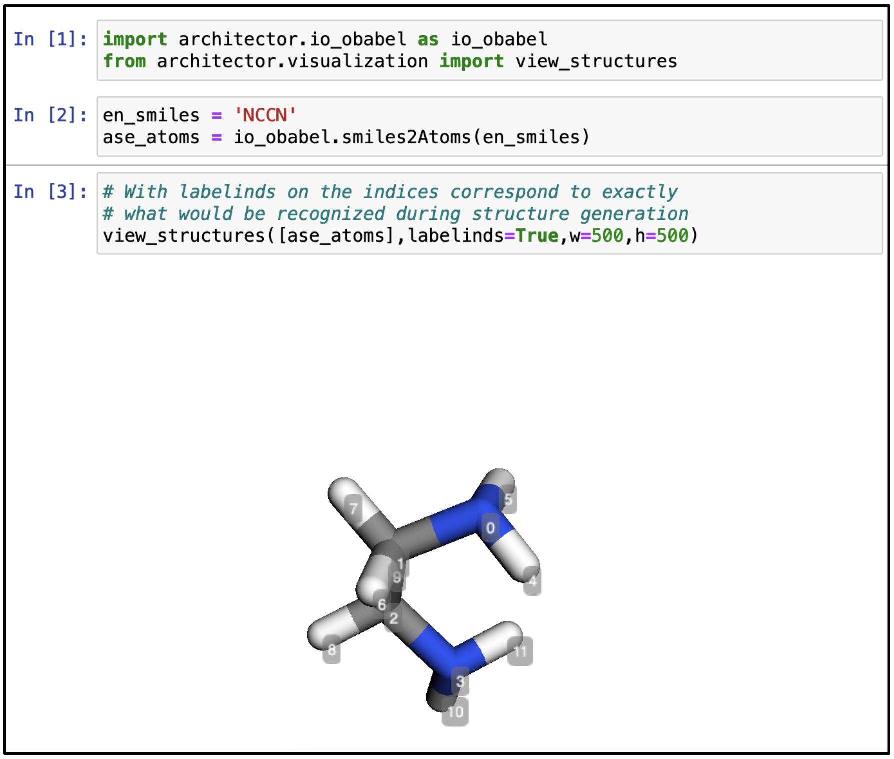

Supplementary Figure 1. Screenshot of an example of *Architector* coordinating atom (CA) determining utility. The SMILES for the ethylenediamine (en) ligand is “NCCN”, which reveals the indices 0 and 3 as the coordinating nitrogen atoms from the ligand. The visualization is performed by the py3Dmol[1] plugin using default jmol colors for all atoms. The utility is readily accessible within the jupyter notebook[2] format installed with *Architector*.

Supplementary Table 1. Default core binding geometries ordered by coordination number (CN). Note that there are 42 default core geometries in total in *Architector* with many derived from common molecular symmetries[3] (e.g. octahedral) supplemented by several CSD-derived molecular symmetries more commonly observed in f-block configurations.

Core CN	Illustration
1	single: 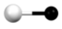
2	bent 109°: 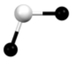 bent 120°: 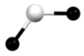 linear: 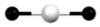
3	T-shaped: 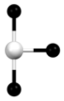 trigonal planar: 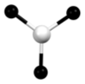 trigonal pyramidal: 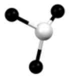
4	seesaw: 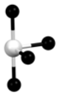 square planar: 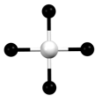 tetrahedral: 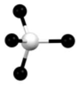
5	pentagonal planar: 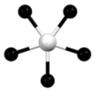 square pyramidal: 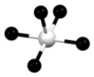 trigonal bipyramidal: 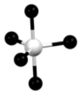
6	hexagonal planar: 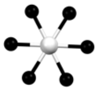 octahedral: 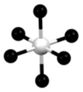 pentagonal pyramidal: 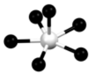 trigonal prismatic: 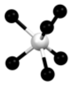
7	Am + 7H_2O: 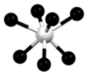 capped octahedral: 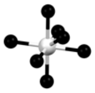 capped trigonal prismatic: 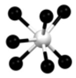 hexagonal pyramidal: 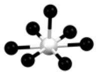 pentagonal bipyramidal: 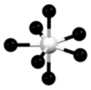
8	axial Bicapped Trigonal Prismatic: 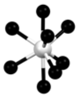 bicapped trigonal Prismatic: 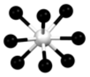 dodecahedron: 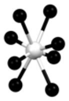 hexagonal Bipyramidal: 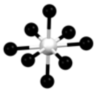 square Prismatic: 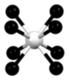 square antiprismatic: 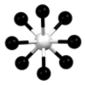 YEMSEP: 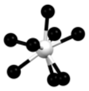

9	Am + $9H_2O$: 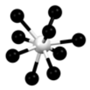 capped square antiprismatic: 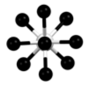 YICLED: 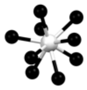 tri-tridentate meridial: 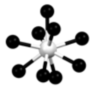 capped trigonal prismatic: 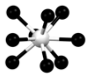
10	axial bicapped hexagonal planar: 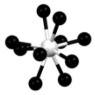 GAKDAY: 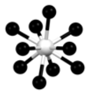 XEGBUJ: 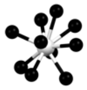
11	cn11_YIJQOX: 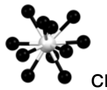 cn11_UTINUI: 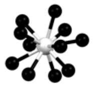 cn11_CABLEW: 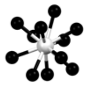
12	penta-bicapped bipyramidal: 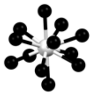 senrag_comp_1: 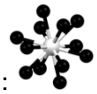

Supplementary Note 1. Ligand classification characteristics.

For reference on ligand geometries across the periodic table we turned to the Cambridge Structural Database (CSD). For each mononuclear metal (M) complex, we removed the metal center in the CSD python API[4] to isolate each ligand. For the ligands we extracted geometric data related to the CA-M vectors and overall ligand bulkiness (Supplementary Figure 2a) to differentiate ligands by shared geometric characteristics for each metal CN. For each ligand a vector was constructed of the normalized CA-M bond distances in descending order of magnitude, all CA-M-CA angles in descending order of magnitude, and the normalized minimum bounding box dimensions in descending order of magnitude was constructed (dist-angle-box vector). For all ligands of a given denticity (e.g. 3) principal component analysis[5] (PCA) was performed. From PCA analysis, clusters in 2D plots of PCA components 1 vs. 2 were manually labeled with chemically relevant tags corresponding to the shared traits of each cluster (Supplementary Figure 2b). Averages of CA-M-CA angles are then used to define each ligand type in further analysis (Supplementary Table 2).

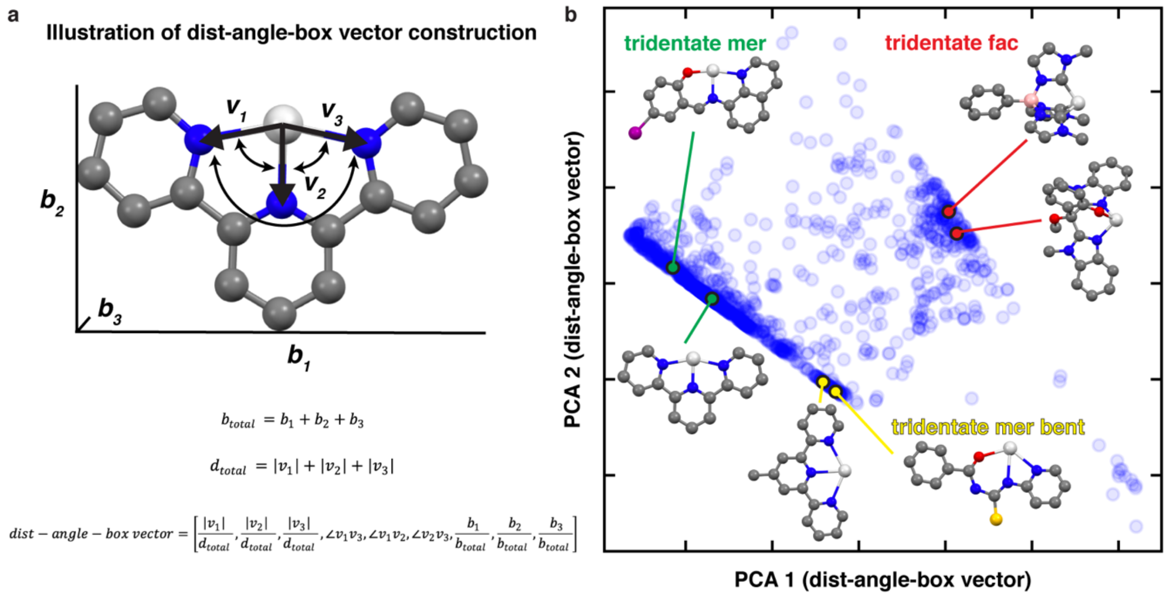

Supplementary Figure 2. **a.** Illustration of example dist-angle-box vector construction for a ligand and **b.** manual labeling of tridentate ligands into "tridentate mer" in green, "tridentate fac" in red, and "tridentate mer bent" geometries via principal component analysis (PCA). Insets show selected examples of ligands from each selected cluster indicating the type of the ligands. Illustration examples are colored with jmol default colors by atom type except for the metal centers which are visually included for reference in silver.

Supplementary Table 2. Default ligand types ordered by denticity. Note that there are 27 default ligand geometries in total in *Architector*. Reported values correspond to mean CA-M-CA angles over all labeled ligand geometries.

		Mean CA-M-CA angle references (•)																														
**ligand type**	**denticity**	**1**	**2**	**3**	**4**	**5**	**6**	**7**	**8**	**9**	**10**	**11**	**12**	**13**	**14**	**15**	**16**	**17**	**18**	**19**	**20**	**21**	**22**	**23**	**24**	**25**	**26**	**27**	**28**	**29**	**30**	**31**
bidentate (bi) cis	2	83.5																														
bi cis bulky	2	82.8																														
bi cischelating	2	50.7																														
bi cis planar	2	79.7																														
bi trans	2	172.8																														
tridentate (tri) mer	3	161.5	82.6	79.5																												
tri fac	3	89.3	86.5	83.9																												
tri mer bent	3	125.7	64.2	61.8																												
tetradentate (tetra) planar	4	177.7	176.6	91.3	90.9	89.7	88.0																									
tetra seesaw	4	162.3	94.0	87.3	84.2	81.8	80.0																									
tetra trigonal pyramidal	4	119.9	116.4	114.4	80.5	79.9	79.4																									
tetra planar bent	4	153.7	136.9	136.0	71.2	68.8	66.6																									
tetra pyramidal	4	111.4	107.7	72.4	71.3	69.6	69.2																									
pentadentate (penta) planar	5	148.4	147.3	142.9	140.9	139.2	77.2	72.8	71.6	70.1	69.0																					
penta square pyramidal	5	175.6	173.3	96.1	94.6	91.8	90.5	89.2	88.0	86.1	83.8																					
penta pyramidal	5	115.7	103.1	100.5	91.6	88.4	61.7	60.3	57.3	56.0	52.1																					
penta planar bent	5	174.2	171.1	125.1	119.5	118.0	115.6	61.0	60.5	59.2	58.3																					
hexadentate (hexa) octahedral	6	175.5	173.1	171.7	97.6	96.1	94.8	93.0	92.2	91.3	89.4	88.7	87.6	84.5	83.2	82.3																
hexa planar	6	174.8	171.4	168.9	122.5	121.4	119.7	118.7	117.5	116.3	62.4	61.6	60.9	60.2	58.8	57.9																
hexa trigonal prismatic	6	153.9	152.6	151.3	116.6	115.2	113.7	87.8	87.3	86.7	86.5	85.9	85.4	79.3	78.8	78.3																
heptadentate (hepta) 5 2	7	157.6	146.0	133.6	129.6	127.7	125.9	122.8	118.3	110.0	89.6	84.6	80.7	77.4	74.0	71.0	66.6	65.9	65.4	64.3	63.4	62.6										
hepta capped trigonal prismatic	7	165.1	164.2	163.6	121.8	121.2	120.8	105.4	104.1	103.5	96.8	96.4	95.8	90.4	90.1	89.7	70.6	70.5	69.7	65.7	65.5	65.4										
hepta pentagonal bipyramidal	7	169.4	148.0	146.9	142.0	138.9	137.0	104.1	100.3	96.4	93.3	90.4	88.8	87.0	85.1	79.4	77.6	75.5	74.3	72.8	70.9	68.7										
octadentate (octa) cubic	8	178.6	173.9	171.8	170.1	114.6	114.0	113.1	112.6	111.7	111.1	108.9	108.4	106.9	106.4	104.6	104.0	82.0	80.5	78.9	68.9	68.3	67.9	67.4	67.1	66.7	66.3	65.8	65.2			
octa trigonal prismatic triface bicapped	8	178.8	140.7	137.1	133.5	123.5	121.3	120.4	120.0	119.6	119.3	118.8	118.2	116.2	101.2	100.2	98.2	97.3	95.0	94.1	61.5	61.0	60.7	60.5	60.3	60.1	59.8	59.5	59.1			
octa square antiprismatic	8	146.3	144.5	141.7	140.7	139.6	138.4	132.5	131.6	130.4	129.4	107.9	102.6	87.3	85.7	84.0	82.1	75.2	74.2	73.4	72.4	69.4	68.9	68.3	67.8	66.8	66.2	65.6	64.8			
nonadentate capped square antiprismatic	9	149.9	147.9	142.5	136.5	136.3	136.1	135.2	134.9	131.8	130.4	127.1	125.4	124.7	122.8	112.5	99.0	95.0	85.9	82.5	77.5	76.6	76.0	74.8	74.2	73.6	72.7	72.5	72.1	68.4	67.6	66.8

```
import pandas as pd
import architector.io_core as io_core
import numpy as np
import architector
from architector.visualization import view_structures
arch_path = '/'.join(architector.__file__.split('/')[:-1])
ligdf = pd.read_csv(arch_path + '/data/angle_stats_datasource.csv')
print('Showing examples of each ligand label!')
ligtypes = ligdf.geotype_label.value_counts().index.values
cns = [ligdf[ligdf.geotype_label == x].cn.values[0] for x in ligtypes]
order = np.argsort(cns)
for i in order:
    print("Ligand label - 'ligType':", "'" + \
          ligtypes[i].replace('_',' ') \
          + "'" + '-' + ' Denticity: ', int(cns[i]))
    tdf = ligdf[ligdf.geotype_label == ligtypes[i]].sample(4)
    view_structures(tdf.xyz_structure,labels=['m']*4,w=100,h=100)
```

```
Showing examples of each ligand label!
Ligand label - 'ligType': 'bi cis'- Denticity:  2
```

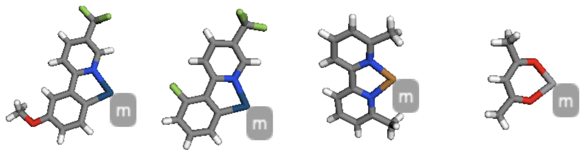

```
Ligand label - 'ligType': 'bi trans'- Denticity:  2
```

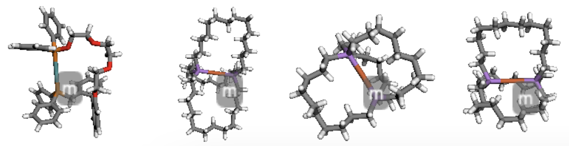

Supplementary Figure 3. Screenshot of example *Architector* ligand type visualization and determination utility. Examples include only the bi cis- and bi trans-type ligands with m indicating the location of the metal and are sampled from the manually labelled ligands from the CSD. The visualization is performed by the py3Dmol plugin using default jmol colors for all atoms. This utility is readily accessible within the jupyter notebook format installed with *Architector*.

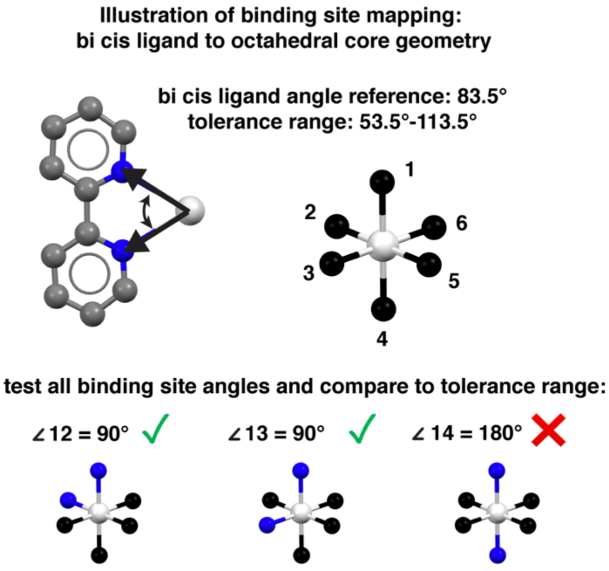

Supplementary Figure 4. Illustration of ligand-core mapping dependent on 30° tolerance range for mapping a bidentate cis ligand to octahedral sites. Below only 3 example sets of bidentate binding sites are shown, but all possible sets binding sites are tested. Black sites indicate potential binding sites at the surface of the core, while blue sites indicate where the ligand would potentially sit.

Supplementary Note 2. Pseudo-energy definition for ranking conformers.

The pseudo-energy for ranking conformers included in *Architector* combines Coulomb repulsion and steric hindrance:

$$\text{Pseudo-energy} = k\left(\sum_{ligand\ pairs\ (i,j)}^{n_{ligand\ pairs}} \frac{q_i q_j (Z_i + Z_j)}{r_{ij}} + \frac{b(Z_i Z_j)}{r_{ij}}\right) \quad \textbf{(Eq 1)}$$

where Z is atomic number and $Z_i = \sum_{atoms\ in\ ligand\ i}^{n_{atoms}} Z_{atom}$, q_i=total elemental charge of ligand i, k is the Coulomb constant (introduced to give output units of energy), b is a constant with units of (elemental charge)^2 , and

$$r_{ij} = \left\| \left(\frac{\sum_{k=1}^{n} v_{k,i}}{\| \sum_{k=1}^{n} v_{k,i} \|}\right) - \left(\frac{\sum_{k=1}^{n} v_{k,j}}{\| \sum_{k=1}^{n} v_{k,j} \|}\right) \right\|_2 \quad \textbf{(Eq 2)}$$

where $v_{k,j}$ is the vector of CA_k assigned to ligand j and n is the total number of CAs assigned to ligand j. The first term in Eq. 1 is designed to encourage conformers with ligands with like charges to be more spatially separated (Coulomb repulsion), while the second is designed to encourage larger ligands to sit further apart (steric hindrance). When mappings correspond to identical values of this pseudo-energy, *Architector* presumes they are symmetrically identical at the metal center, and only one mapping from each set is preserved.

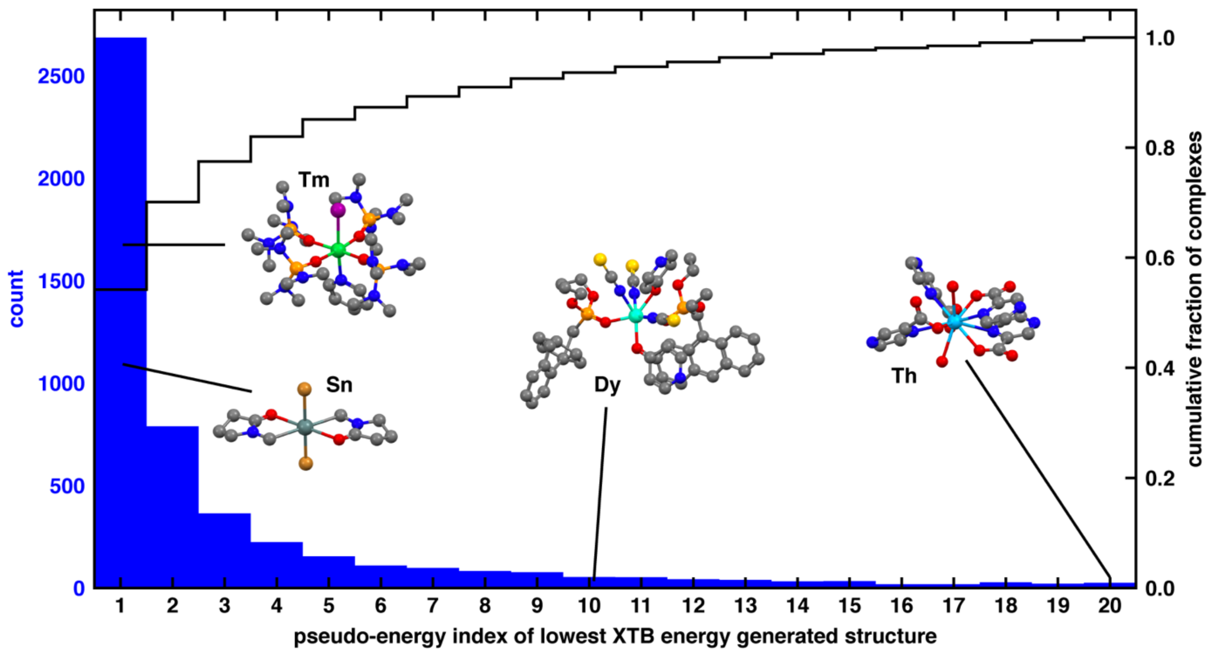

Supplementary Figure 5. Histogram of pseudo-energy index (e.g. 1 indicates the lowest pseudo-energy prediction, 2 indicates next-lowest etc.) of the lowest-*xTB* energy *Architector* generated structures. These generations requested building the top 20 lowest pseudo-energy predicted structures from over 4,596 distinct structures. Cumulative fractions of complexes values are 0.936 at pseudo-energy index = 10 and 0.995 at pseudo-energy index = 20, indicating ~94% of lowest-energy configurations are sampled within the top 10, and nearly all are sampled within the top 20. Inset examples are *Architector*-generated structures, highlighting the increasing metal-center complexity moving from pseudo-energy index = 1 to pseudo-energy index = 20. All elements in the inset renderings are colored by default jmol colors.

Supplementary Table 3. Distance Matrix Construction. All covalent radii (r_{cov}) are defined from single-bond covalent radii literature[6] while Van der Waal radii (r_{vdw}) are from literature.[7] ∠CA-M-CA angles are defined from core binding sites assigned to the ligands. Note that these distances and cutoffs were largely heuristically determined to allow for both the flexibility in binding positions and to constrain to somewhat reasonable geometries. In this respect, the objective is to enable the generation of conformers which can be optimized to varied and valid 3D configurations up to high denticity ligands, in contrast to directly generating low-energy conformations.

Type of Pair involved in Distance (1-2)	Lower Bound (LB)	Upper Bound (UB)	Comment
M-CA	0.8*($r_{cov,1} + r_{cov,2}$)	1.2*(same as LB)	
CA-CA	0.9* $\sqrt[2]{\left(r_{cov,CA1} + r_{cov,M}\right)^2 + \left(r_{cov,CA2} + r_{cov,M}\right)^2 - 2 * \left(r_{cov,CA1} + r_{cov,M}\right) * \left(r_{cov,CA2} + r_{cov,M}\right) * cos\,(\angle CA_1 - M - CA_2)}$	1.1*(same as LB)	Cosine rule
M-CA neighbor (non-hydrogen)	1.2*($r_{cov,1} + r_{cov,2}$)	20 Å	Encourage conformations with less M crowding
M-CA neighbor (hydrogen)	1.1*($r_{vdw,1} + r_{vdw,2}$)	20 Å	Allow hydrogens slightly closer to the metal center
M-Ligand Atom < depth 4 graph steps from M (non-hydrogen)	1.3*($r_{vdw,1} + r_{vdw,2}$)	50 Å	Encourage less M crowding
M-Ligand Atom < depth 4 graph steps from M (non-hydrogen)	1.1*($r_{vdw,1} + r_{vdw,2}$)	50 Å	
M (Z ≥ 57)-Ligand Atom ≥ depth 4 graph steps from M (non-hydrogen)	1.2*($r_{vdw,1} + r_{vdw,2}$)	100 Å	For lanthanides and actinides, allow smaller interaction distance due to larger radii
M (Z < 57)-Ligand Atom ≥ depth 4 graph steps from M (non-hydrogen)	1.5*($r_{vdw,1} + r_{vdw,2}$)	100 Å	
M-Ligand Atom ≥ depth 4 graph steps from M (hydrogen)	1.3*($r_{vdw,1} + r_{vdw,2}$)	100 Å	
Ligand-Ligand neighbors	0.9*(initial ligand conformer distance)	1.1*(same as LB)	
Ligand-ligand next nearest neighbors	0.9*(initial ligand conformer distance)	1.1*(same as LB)	
Other ligand-ligand atom pairs	($r_{vdw,1} + r_{vdw,2}$)	100 Å	Allow ligand flexibility away from local environment

Supplementary Note 3. Distance geometry application.

Briefly, as specified in Supplementary Table S3 we construct upper-bound and lower-bound distance matrices from ± 30% tolerances on initial distances from the forcefield (FF)-relaxed geometries for neighbors and next nearest neighbors. M-CA distances are set with ± 30% tolerances from their respective single-bond covalent radii (r_{cov}),[6] while CA-CA distances are set from the M-CA distances along with CA-M-CA angles set by the binding sites. Distances for atoms more than 2 steps away on the molecular graph are set from a lower bound corresponding to the sum of their van der waals (vdw) radii[7] to an upper bound of 100Å to allow the flexibility as is needed for cases with multidentate metal-ligand bonding. From here, triangle smoothing and partial metrization[8] is performed, followed by

conjugate-gradient minimization of positions, target distance errors, and distance-error gradients to generate a ligand conformer.[8,9] If the partial metrization fails to embed the conformer in 3D, the embedding is attempted up to 600 times with incrementally loosened upper and lower bounds constraints.

Supplementary Note 4. Forcefield cleaning and CA reassignment.

To clean the distance-geometry generated structures with FFs we perform 4 sequential steps: 1. UFF[10] relaxation without angle constraints 2. Reintroduce hydrogens which were removed during distance geometry sampling and re-relax with UFF, keeping the metal atom fixed 3. Reorder multidentate (>2) ligand CAs to minimize deviations in angles between the assigned sites and chemically informed UFF-relaxed sites [11,12] 4. UFF relaxation with constraints imposed on CA-M-CA bonds to ensure closest agreement between reassigned CAs and core geometry vectors and 5. Delete metal atom and re-relax with MMFF94[13] where applicable, keeping CA atom positions fixed.

Supplementary Note 5. Kabsch algorithm alignment of ligand to core binding sites.

Before rotation to maximize agreement with assigned core binding sites, the ligands are positioned with the metal center at the origin, defining CA-M vectors to the origin. Kabsch rotation is performed to calculate the rotation that closely matches the CA-M vectors to the core binding site vectors.[14] Since the ligand conformations are generated without specific binding site identities as constraints (i.e. using only CA-M-CA angles) there is no guarantee the chirality of the generated ligand and binding sites will match the chirality of the assigned core binding sites. Therefore, for denticity>2 ligands, Kabsch alignment and rotational loss is calculated for both the ligand and its x-plane mirror, and the lowest rotational loss is assigned as the correct rotation. The rotational root mean squared deviation (rotRMSD) between the rotated CA-M vectors and the assigned core binding sites is defined from the Kabsch algorithm implementation in scipy.[12]

Supplementary Note 6. Interatomic distance sanity checks.

Interatomic distance sanity checks are designed to address three common modes of failure during assembly or after final relaxation: (1) Overlapping atoms (2) Isolated atoms and (3) Structures deviated from requested molecular graphs. To address all 3, a distance matrix is first constructed from all interatomic distances for the molecule of interest:

$$DistMat(structure) = \begin{bmatrix} 0 & |atom_{1,position} - atom_{2,position}| & \cdots \\ |atom_{2,position} - atom_{1,position}| & 0 & \cdots \\ \vdots & \vdots & \ddots \end{bmatrix}$$

Where $atom_{i,position}=[atom_{i,x},atom_{i,y},atom_{i,z}]$. Additionally, a reference distance matrix is constructed using the sum of the covalent radii:

$$RefDistMat(structure) = \begin{bmatrix} 0 & r_{cov,atom1} + r_{cov,atom2} & \cdots \\ r_{cov,atom2} + r_{cov,atom1} & 0 & \cdots \\ \vdots & \vdots & \ddots \end{bmatrix}$$

Where $r_{cov,I}$ is the reference covalent radii[5] of atom i. Finally, for failure mode (3), the desired molecular graph is represented a matrix:

$$MolecularGraph(structure) = \begin{bmatrix} 0 & 1 & \dots \\ 1 & 0 & \dots \\ \vdots & \vdots & \ddots \end{bmatrix}$$

Where 1 indicates a desired bond and 0 indicates no bond between the atoms of the row index and the column index.

From these matrices we can check all 3 failure modes. To rule out failure mode (1), if any off-diagonal elements of the DistMat are less than the same element in the Factor1*RefDistMat (Factor1 is usually around 0.5) the structure is flagged. For failure mode (2) we check that for each atom i in the structure, the largest off-diagonal elements in the DistMat are less than Factor2, which is usually around 4 Å. Finally, for failure mode (3) we check if any non-zero elements of the MolecularGraph in the DistMat are greater than Factor3*RefDistMat (Factor 3 is usually around 1.5). Default numbers employed for Factors1, 2, and 3 are detailed in Supplementary Table S4.

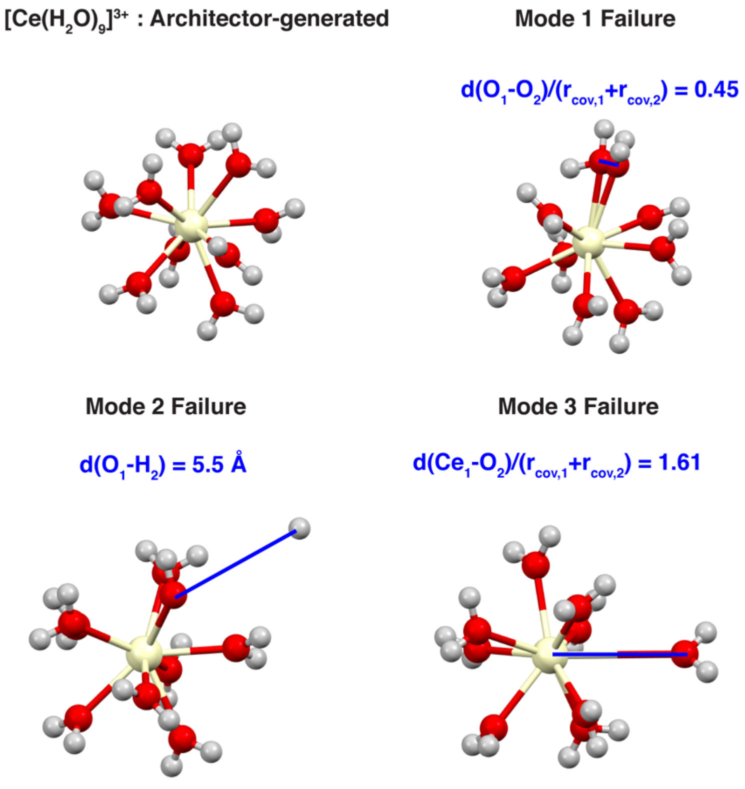

Supplementary Figure 6. Illustration of "chemically sane" structure (upper left) generated from *Architector* and examples of all three failure modes. Mode 1 Failure (upper right) shows an example where waters have been placed on top of each other, resulting in excessively short interatomic distances. Mode 2 failure shows a case where a hydrogen atom has moved away, resulting in an isolated atom where its minimum distance is 5.5 Å. Finally, Mode 3 failure shows the elongated Ce-O bond. The illustrated structures have one and only one of each failure mode. The insets are colored by jmol colors.

Supplementary Table 4. Default "chemically sane" factors. Note that final sanity evaluation factors are stricter than assembly evaluation factors. Note that 1.8 is used for alkali and alkaline earth metals due to their relative propensity for ionic bonds, which tend to be longer than covalent bonds, which are referenced in the calculation of mode of failure 3.

Architector Step, Chemistry	Factor1	Factor2 (Å)	Factor3
Assembly, All types	0.3	4	1.8
Final Evaluation, transition metals, post-transition metals, lanthanides, actinides	0.55	3.5	1.7
Final Evaluation, alkali metals, alkaline earth metals	0.55	3.5	1.8
DFT Evaluation Filter	0.8	3	1.4

```
import itertools
from random import sample
from architector import (build_complex,
                         view_structures,
                         convert_io_molecule,
                         smiles2Atoms)
inputDict = {'core':{'metal':'Fe','coreType':['octahedral']}, # Specify metal center geometry as octahedral
            'ligands':[{'smiles':'n1ccccc1-c2ccccp2','coordList':[0,11],'ligType':'bi_cis'}]*3,
            'parameters':{'return_full_complex_class':True, # Needed to get back full complex class
                          'save_init_geos':True, # Save inital geometries
                          'return_only_1':True}} # Save only 1 for speed
arch_out = build_complex(inputDict); # Now just build using the dictionary!
print('xTB-relaxed Architector Structure:')
view_structures(arch_out)
comp_class = arch_out[list(arch_out.keys())[0]]['full_complex_class'] # Pull out the conformer
def all_initial_conformers(comp_class):
    ligs = []
    for i,lig in enumerate(comp_class.__dict__['ligandList']):
        ligs.append(lig.__dict__['conformerList'])
    out_conformers = []
    for things in itertools.product(*ligs):
        ats = smiles2Atoms(comp_class.coreSmiles)
        ats.set_positions([(0,0,0)])
        for item in things:
            ats += item
        out_conformers.append(ats)
    return out_conformers
out = all_initial_conformers(comp_class)
print('Total Number of Conformers',len(out))
samp = sample(out,20)
print('Different Metal/Ligand Conformer Architector Structures:')
view_structures(samp)
```

```
xTB-relaxed Architector Structure:
```

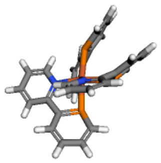

```
Total Number of Conformers 1728
Different Metal/Ligand Conformer Architector Structures:
```

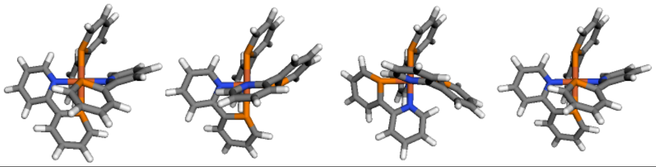

Supplementary Figure 7. Code and visualization of iteration of all different possible metal-ligand conformers mapped to the metal center.

Supplementary Table 5. *Architector* default oxidation, metal spin, and coordination numbers (CNs).

Chemical Symbol	Atomic Number	Default Oxidation State	Default Spin (number of unpaired electrons)	Default Core CNs
Li	3	1	0	[4,6,8]
Be	4	2	0	[4,6,8]
Na	11	1	0	[4,6,8]
Mg	12	2	0	[4,6,8]
Al	13	3	0	[4,6]
K	19	1	0	[4,6,8]
Ca	20	2	0	[4,6,8]
Sc	21	3	0	[4,6]
Ti	22	4	0	[4,6]
V	23	5	0	[4,6]
Cr	24	3	3	[4,6]
Mn	25	2	5	[4,6]
Fe	26	2	4	[4,6]
Co	27	2	3	[4,6]
Ni	28	2	2	[4,6]
Cu	29	2	1	[4,6]
Zn	30	2	0	[4,6]
Ga	31	3	0	[4,6]
Rb	37	1	0	[4,6,8]
Sr	38	2	0	[4,6,8]
Y	39	3	0	[4,6]
Zr	40	4	0	[4,6]
Nb	41	5	0	[4,6]
Mo	42	6	0	[4,6]
Tc	43	5	2	[4,6]
Ru	44	2	4	[4,6]
Rh	45	1	2	[4,6]
Pd	46	2	0	[4,6]
Ag	47	1	0	[4,6]
Cd	48	2	0	[4,6]
In	49	3	0	[4,6]
Sn	50	4	0	[4,6]
Cs	55	1	0	[4,6,8]
Ba	56	2	0	[4,6,8]
La	57	3	0	[7,8,9]
Ce	58	3	1	[7,8,9]
Pr	59	3	2	[7,8,9]
Nd	60	3	3	[7,8,9]
Pm	61	3	4	[7,8,9]
Sm	62	3	5	[7,8,9]
Eu	63	3	6	[7,8,9]
Gd	64	3	7	[7,8,9]
Tb	65	3	6	[7,8,9]
Dy	66	3	5	[7,8,9]
Ho	67	3	4	[7,8,9]
Er	68	3	3	[7,8,9]
Tm	69	3	2	[7,8,9]
Yb	70	3	1	[7,8,9]
Lu	71	3	0	[7,8,9]
Hf	72	4	0	[4,6]
Ta	73	5	0	[4,6]
W	74	6	0	[4,6]
Re	75	4	1	[4,6]
Os	76	2	0	[4,6]
Ir	77	3	0	[4,6]
Pt	78	2	0	[4,6]
Au	79	3	2	[4,6]
Hg	80	2	0	[4,6]
Tl	81	3	0	[4,6]
Pb	82	2	0	[4,6]
Bi	83	3	0	[4,6]
Fr	87	1	0	[4,6,8]
Ra	88	2	0	[4,6,8]
Ac	89	3	0	[6,7,8,9]
Th	90	4	0	[6,7,8,9]
Pa	91	5	0	[6,7,8,9]
U	92	4	2	[6,7,8,9]
Np	93	4	3	[6,7,8,9]
Pu	94	4	4	[6,7,8,9]
Am	95	3	6	[6,7,8,9]
Cm	96	3	7	[6,7,8,9]
Bk	97	3	6	[6,7,8,9]
Cf	98	3	5	[6,7,8,9]
Es	99	3	4	[6,7,8,9]
Fm	100	3	3	[6,7,8,9]
Md	101	3	2	[6,7,8,9]
No	102	2	0	[6,7,8,9]
Lr	103	3	0	[6,7,8,9]
Rf	104	3	1	[4,6]
Db	105	5	0	[4,6]
Sg	106	6	0	[4,6]
Bh	107	7	0	[4,6]
Hs	108	8	0	[4,6]
Nh	113	1	0	[4,6]
Fl	114	2	0	[4,6]
Mc	115	1	2	[4,6]
Lv	116	2	2	[4,6]

Supplementary Note 7. Assigning default spin, charge state, and core coordination number (CN) to assembled complexes.

If neither a spin nor charge state is assigned to a complex or metal, the default values from Supplementary Table S5 are used to calculate and assign spin and charge. The complex total charge is calculated by:

$$Q_{total} = q_{metal} + \sum_{i=1}^{n} q_{ligand,i}$$

Where n is the total number of ligands. $q_{ligand,i}$ is calculated from the input SMILES and Open Babel[15] -determined total charge of the ligand. After the total charge is assigned, whether there is an even or odd number of electrons in the complex is determined with 4 different methods: (1) If there is an even number of electrons and an even metal spin requested, the spin of the whole complex is set to the metal spin. (2) If there is an even number of electrons and an odd metal spin requested, the total spin of the complex is set to the metal spin -1. (3) If there is an odd number of electrons in the complex and a metal spin requested <2 the total spin of the complex is set to either 1 or 0 depending on if the metal spin is 0 or 1, respectively. (4) If there is an odd number of electrons in the complex and a metal spin requested <7 the total spin is set to the metal spin +1, while if the metal spin requested is >6 the total spin is set to the metal spin -1. Thus, complexes with low, medium, and high spin are attempted to be preserved by default.

For complexes without an assigned core CN, all default CN values are attempted. For core CNs where the ligands do not fully fill the core CN, water molecules are added as "fill ligands" by default in order to fill out the core CN. The identity of the "fill ligand" can be specified as a parameter before construction in cases where other ligands are desired.

Supplementary Note 8. Additional details utilized during complex construction.

Several intermediate steps are implemented during complex construction beyond those described in the manuscript. For full reference on additional details, we suggest consulting the *Architector* codebase at github.com/lanl/Architector.

First, for structures, different numbers of ligand conformers are sampled depending on the denticity of the ligands and symmetry of metal-ligand atom interactions. For most ligands, 6 conformers will be generated, 3 with different random states for conformer generation, 1 with another random state for generation but with no MMFF relaxation after UFF relaxation, and 2 with all M-L atom distance upper bounds all set to 100 angstroms to encourage uncrowded conformers. Exceptions are added to sample greater symmetries of ligands surrounding the metals. For example, one exception to this rule is monodentate ligands, where 3 additional ligand conformers are added corresponding to (a.) the first successfully generated ligand conformer aligned directly to the coordination site, (b.) (a.) rotated by 45 degrees around the coordination site vector, and (c.) (a.) Rotated by 90 degrees around the coordination site. This helps sample the configurational/rotational space of the ligands surrounding the metal center. Other exceptions are made for bidentate-cis, tridentate-meridial and -facially bound ligands where rotations of ligands around the coordination sites are well-defined.

Second, for most ligand structures, during distance geometry generation of conformers and initial UFF relaxation of conformers, the hydrogens are removed and then re-added utilizing the underlying openbabel utility.

This is done to dramatically reduce the dimensionality required for solving the distance geometry equations and generally results in more sensible conformers than when hydrogens are included in the distance matrices in distance geometry.

Third, currently there are no stereochemistry constraints imposed on ligand conformers. This was done to encourage physically relevant conformers for systems with complex metal-center stereochemistries but may generate structures different from those desired for applications other than energetic evaluation. Incorporation of ligand stereochemistry should be possible in later iterations of the *Architector* codebase.

Supplementary Note 9. Mining the CSD for mononuclear complexes.

To obtain all the mononuclear complexes from the CSD and retrieve the associated ligand smiles and coordinating atoms, a Conquest query[16] was performed for any metal atom bound to 4 or more atoms was performed. From the produced list of potential structures, the CSD python API[4] was used to iterate over all structures and isolate components (representing distinct molecular sub-graphs of the full crystal structures) from each x-ray crystallographic diffraction (XRD)-determined structure that contain 1 and only 1 metal, indicating mononuclear complexes. Metals are defined by all boxed elements in Figure 1 and Figure S7. For each mononuclear component the python API was used to add missing hydrogens, and then each complex in addition to its CSD Refcode and component index in the XRD structure, including the molecular graph defined by the CSD, are saved in the MOL2 file format.

For each complex, the metal center was removed, and the remaining ligands were converted to SMILES strings from the Python API, and the ligand graph containing the information of which atoms were coordinating to the metals saved.[17] Further checks were performed to remove invalid structures (Supplementary Table S6): 1. Hydrogen Mismatch or Error cases were flagged - where the CSD api added additional hydrogens when requested indicated undersaturated compounds. 2. Small interatomic distances cases where the interatomic distance is less than 0.6*(sum of single-bond covalent radii[6]) and all interatomic angles as defined by the CSD-labeled molecular graph are checked to be greater than 29°. 3. Haptic ligands with an additional coordinating atom (CA) and Edge-bound ligands (ligands with neighbors that are both coordinating the metal center) are currently unsupported in *Architector* construction.

Additionally, 4. For some complexes the SMILES conversion fails internally in the CSD python API, largely due to disordered structures. 5. For some ligands the SMILES with CAs molecular graph do not match the CSD 3D structure, meaning comparing these structures via *Architector* is not possible. To perform this the SMILES to coordinating atom mapping we used pyNauty graph matching with the molecular graphs with vertices colored by atomic numbers.[18] 6. To check for validity of the coordinating atom assignments we compared the molecular graphs using both the full 3D structure from the CSD and the one imposed by the SMILES and CA labels to ensure both encoded the same metal-ligand pair. 7. Metals with CN > 12 were filtered to reduce additional complexity for high-throughput and represent only a very minimal number of structures (2 structures).

Further checks were performed on total charge and oxidation state. Both were interpreted from CSD user inputs across the complexes and verified against assigned CSD python api-interpreted ligand charges. 7. Complexes with total charges less that -2 and greater than 4 were removed to due to these complexes likely being formed in highly charged crystalline lattices, making molecular electronic structure methods less reliable. 8. oxidation states assigned

less that 1 and greater that 7 were removed due to their relative lack of abundance in the CSD and likelihood of charge assignment error. For all metals with the exception of the actinides, the oxidation states selected were required to be in the Mendeleev[19] package's list of common oxidation states. For actinides, we allowed for all oxidation states in the range of (III)-(VI). 9. As a final check, we verified that the full complex definition from all ligands and the metal center encoded the same molecular graph, ensuring a match between the assembled complexes from the 2D graph information. 10. Lastly, we selected one duplicate from each set of complexes with matching molecular graph determinants, oxidation states, and total charges to eliminate the possibility of reproducing copies of identical complexes with *Architector*.[20]

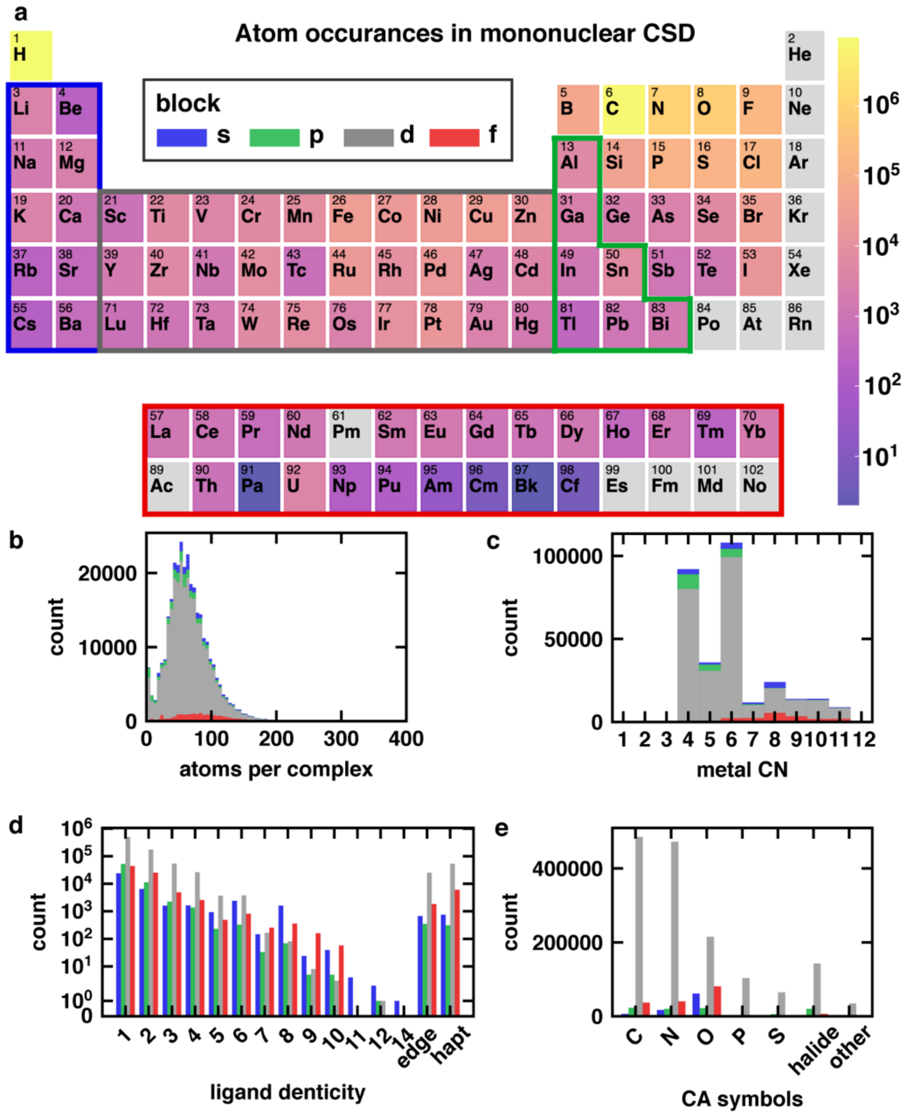

Supplementary Figure 8. Distributions across the full set of mononuclear complexes in the CSD. **a.** Atom distributions across the full CSD as colored on the periodic table. **b.** Stacked histogram of the number of atoms per complex. **c.** stacked histogram of the metal coordination number (CN) **d.** Log-normed histogram of the ligand types bound to the metal centers. **e.** metal-coordinating atom (CA) symbols. Note the prevalence of d-block complexes, and in especially b-d that f-block complexes tend to have more atoms per complex, higher coordination number, and higher ligand denticities. Additionally, note that *Architector* only supports ligand denticities up to 9 and haptic ligands (hapt). Edge bound ligands are ligands where atoms that are neighbors are bound to the metal centers.

Supplementary Table 6. Mononuclear CSD filtering steps to get *Architector*-compatible structures. From top to bottom these are the filters applied to the mononuclear CSD to get the pool of potential complexes to replicate.

**Total CSD:**	312,527	8,
**Filter Reason**	**Remaining**	**Removed**
Hydrogens Mismatch or Error	285,922	26,605
Small interatomic distances or Angles < 29°	281,239	4,683
Haptic Ligands with additional CA (Not currently supported by *Architector*)	277,531	3,708
Edge-bound ligands (Not currently supported by *Architector*)	257,436	20,095
CSD SMILES conversion error	228,033	29,403
SMILES with CA labels do not match 3D structure stoichiometry	217,098	10,935
Metal CN > 12 (Very limited number of structures)	217,096	2
5 > Total Charges > -3	165,869	51,227
0 < Oxidation State <8 and in common oxidation state list	160,512	5,357
Complex molecular graph mismatches	157,118	3,394
Remove Duplicates in matching graph, oxidation state, and charge state	**113,630**	43,488

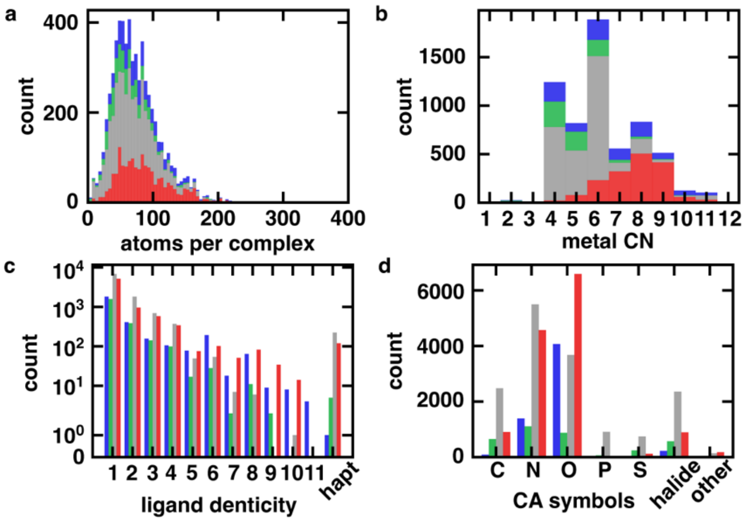

Supplementary Figure 9. Distributions across the sampled *Architector*-compatible mononuclear complexes in the CSD. **a.** Stacked histogram of the number of atoms per complex. **b.** stacked histogram of the metal coordination number (CN) **c.** Log-normed histogram of the ligand types bound to the metal centers. **d.** metal-coordinating atom (CA) symbols. Note that d-block complexes are much less prevalent, and f-block complexes are larger in terms of atoms per complex, and more highly coordinated, and have more of the higher denticity ligands than the s-,p-, or d-block complexes. Additionally, f-block metals tend to be coordinated more frequently by O and N than the d-block complexes.

Supplementary Table 7. *Architector* replication set oxidation state and spin state counts.

Chemical Symbol	Atomic Number	Oxidation State(s)	Metal Spin(s) (number of unpaired electrons)	Count(s)
Li	3	1	0	100
Be	4	2	0	100
Na	11	1	0	100
Mg	12	2	0	100
Al	13	3	0	100
K	19	1	0	100
Ca	20	2	0	100
Sc	21	3	0	100
Ti	22	4,3,2	0,1,2	61,20,19
V	23	4,5,3,2	1,0,2,3	44,38,11,7
Cr	24	3,2,6	3,4,0	72,27,1
Mn	25	2,3,4,6,7	5,4,3,1,0	70,23,5,1
Fe	26	2,3	4,5	69,31
Co	27	2,3	3,4	64,36
Ni	28	2	2	100
Cu	29	2,1	1,0	78,22
Zn	30	2	0	100
Ga	31	3	0	100
Rb	37	1	0	62
Sr	38	2	0	100
Y	39	3	0	100
Zr	40	4	0	100
Nb	41	5	0	100
Mo	42	6,4	0,2	75,25
Tc	43	4,7	3,0	25,15
Ru	44	3,4	5,4	68,32
Rh	45	3	4	100
Pd	46	2,0,4	2,0,4	97,2,1
Ag	47	1	0	100
Cd	48	2	0	100
In	49	3	0	100
Sn	50	4,2	0,0	91,9
Cs	55	1	0	52
Ba	56	2	0	100
La	57	3	0	100
Ce	58	3,4	1,0	69,31
Pr	59	3	2	100
Nd	60	3	3	100
Sm	62	3	5	100
Eu	63	3,2	6,7	85,15
Gd	64	3	7	100
Tb	65	3	6	100
Dy	66	3	5	100
Ho	67	3	4	100
Er	68	3	3	100
Tm	69	3	2	82
Yb	70	3	1	100
Lu	71	3	0	100
Hf	72	4	0	100
Ta	73	5	0	100
W	74	6,4	0,2	67,33
Re	75	7,4	0,3	54,46
Os	76	4	4	100
Ir	77	3,4	4,5	97,3
Pt	78	2,4	2,4	86,14
Au	79	3,1	2,0	88,12
Hg	80	2	0	100
Tl	81	3,1	0,0	82,18
Pb	82	2,4	0,0	85,15
Bi	83	3	0	100
Th	90	4	0	100
Pa	91	5	0	1
U	92	6,4,3,5	0,2,3,1	45,37,9,9
Np	93	5,4,6,3	2,3,1,4	22,18,9,3
Pu	94	4,3,6	4,5,2	24,18,8
Am	95	3	6	8
Cm	96	3	7	2
Bk	97	3	6	2
Cf	98	3	5	3

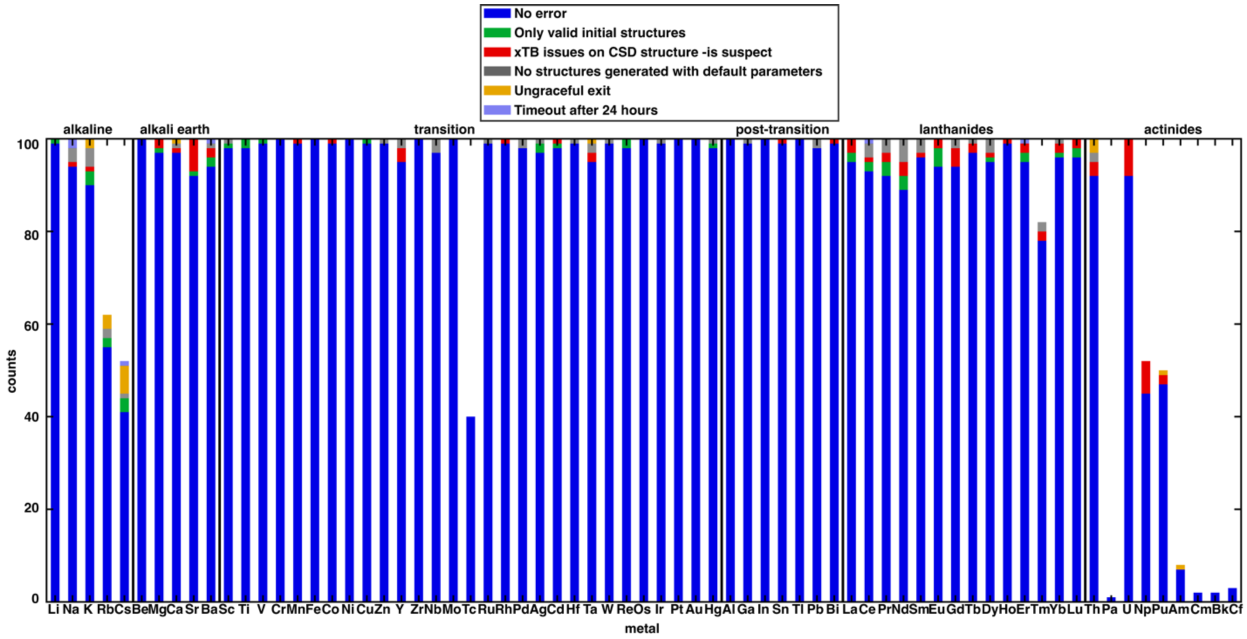

Supplementary Figure 10. Stacked bar chart highlighting distribution of types of successful and unsuccessful generations across the periodic table. “Only valid initial structures” are structures where the generated geometry became distorted during *xTB* relaxation or failed during the relaxation. “xTB issues on CSD structure - is suspect” successfully generated with *Architector*, but the CSD structure distorted or failed during relaxation. “No structures generated with default parameters” did not output any structures during *Architector* generation – constituting failures of *Architector* to generate a structure. “Ungraceful exit” calculations consist of calculations that resulted in a crash of *xTB* during either complex generation or during CSD relaxation. “Timeout after 24 hours” were stopped after 24 hours of generation.

Supplementary Table 8. Numbers of calculations complete and counts of errors and/or missing by type.

		"Unsuccessful" Complex Generations						
**metals**	**successful complex generations**	**only valid initial structures**	**xTB issues on CSD - structure is suspect**	**no structures generated with default parameters**	**ungraceful exit**	**timeout (24 hours)**	**total attempted**	**successful/ (total attempted)**
Li	99	1	0	0	0	0	100	0.99
Na	94	0	1	3	2	0	100	0.94
K	90	3	1	4	2	0	100	0.90
Rb	55	2	0	2	3	0	62	0.89
Cs	41	3	0	1	6	1	52	0.79
Be	100	0	0	0	0	0	100	1.00
Mg	97	1	2	0	0	0	100	0.97
Ca	97	0	1	1	1	0	100	0.97
Sr	92	1	7	0	0	0	100	0.92
Ba	94	2	2	1	0	1	100	0.94
Sc	98	1	0	1	0	0	100	0.98
Ti	98	2	0	0	0	0	100	0.98
V	99	1	0	0	0	0	100	0.99
Cr	100	0	0	0	0	0	100	1.00
Mn	99	0	1	0	0	0	100	0.99
Fe	100	0	0	0	0	0	100	1.00
Co	99	0	1	0	0	0	100	0.99
Ni	100	0	0	0	0	0	100	1.00
Cu	99	1	0	0	0	0	100	0.99
Zn	99	0	0	1	0	0	100	0.99
Y	95	0	3	2	0	0	100	0.95
Zr	100	0	0	0	0	0	100	1.00
Nb	97	0	0	3	0	0	100	0.97
Mo	100	0	0	0	0	0	100	1.00
Tc	40	0	0	0	0	0	40	1.00
Ru	99	0	0	1	0	0	100	0.99
Rh	99	0	1	0	0	0	100	0.99
Pd	98	0	0	2	0	0	100	0.98
Ag	97	2	0	1	0	0	100	0.97
Cd	98	1	1	0	0	0	100	0.98
Hf	99	0	0	1	0	0	100	0.99
Ta	95	0	2	2	1	0	100	0.95
W	99	0	0	1	0	0	100	0.99
Re	98	2	0	0	0	0	100	0.98
Os	100	0	0	0	0	0	100	1.00
Ir	99	0	0	1	0	0	100	0.99
Pt	100	0	0	0	0	0	100	1.00
Au	100	0	0	0	0	0	100	1.00
Hg	98	1	0	1	0	0	100	0.98
Al	100	0	0	0	0	0	100	1.00
Ga	99	0	0	1	0	0	100	0.99
In	100	0	0	0	0	0	100	1.00
Sn	99	0	1	0	0	0	100	0.99
Tl	100	0	0	0	0	0	100	1.00
Pb	98	0	0	2	0	0	100	0.98
Bi	99	0	1	0	0	0	100	0.99
La	95	2	3	0	0	0	100	0.95
Ce	93	2	1	3	0	1	100	0.93
Pr	92	3	2	3	0	0	100	0.92
Nd	89	3	3	5	0	0	100	0.89
Sm	96	0	1	3	0	0	100	0.96
Eu	94	4	2	0	0	0	100	0.94
Gd	94	0	4	2	0	0	100	0.94
Tb	97	0	2	1	0	0	100	0.97
Dy	95	1	1	3	0	0	100	0.95
Ho	99	0	1	0	0	0	100	0.99
Er	95	2	2	0	0	1	100	0.95
Tm	78	0	2	2	0	0	82	0.95
Yb	96	1	2	1	0	0	100	0.96
Lu	96	2	2	0	0	0	100	0.96
Th	92	0	3	2	3	0	100	0.92
Pa	1	0	0	0	0	1	1	1.00
U	92	0	8	0	0	0	100	0.92
Np	45	0	7	0	0	0	52	0.87
Pu	47	0	2	0	1	0	50	0.94
Am	7	0	0	0	1	0	8	0.88
Cm	2	0	0	0	0	0	2	1.00
Bk	2	0	0	0	0	0	1	1.00
Cf	3	0	0	0	0	0	2	1.00
**Mean**	86.32	0.64	1.06	0.83	0.26	0.09	89.19	**0.97**
**St Dev**	27.25	1.01	1.69	1.18	0.92	0.33	27.46	0.04
**Min**	1	0	0	0	0	0	1	0.79
**Max**	100	4	8	5	6	2	100	1
**Total**	5956	44	73	57	18	6	**6154**	
	**Fraction of "Unsuccessful"**	0.22	0.37	0.29	0.09	0.03		

Supplementary Note 10. Describing unsuccessful generation modes of failure.

The most common source of unsuccessful generation ("xTB issues on CSD - structure is suspect", 73 structures) was where the initial structures generated from *Architector* were able to be evaluated, but the *xTB* evaluation over a geometry relaxation on the CSD structure itself gave errors or converged to distorted structures by the sanity checks described above. The next most common source of unsuccessful generation ("no structures generated with default parameters", 57 structures) was where *Architector* was unable to generate any valid conformers due to an inability to map the ligand types to the cores tested from binding sites or in 3D, due to either bulky ligands or crowded metal centers, constituting the only real "failures" of *Architector* to produce a structure. In some of these cases, structures could be generated with looser "chemical sanity" parameters. In other cases, *Architector* generated valid initial conformers by according to the sanity checks described above, but *xTB* failed to converge geometry relaxation on all generated *Architector* structures ("only valid initial structures", 44 structures). Finally, only a small percentage of unsuccessful generations (18 structures, 9%) resulted in a processing error from *xTB* ("ungraceful xTB exit") or timed out during generation (> 24 hours, 6 structures) due to larger complex sizes.

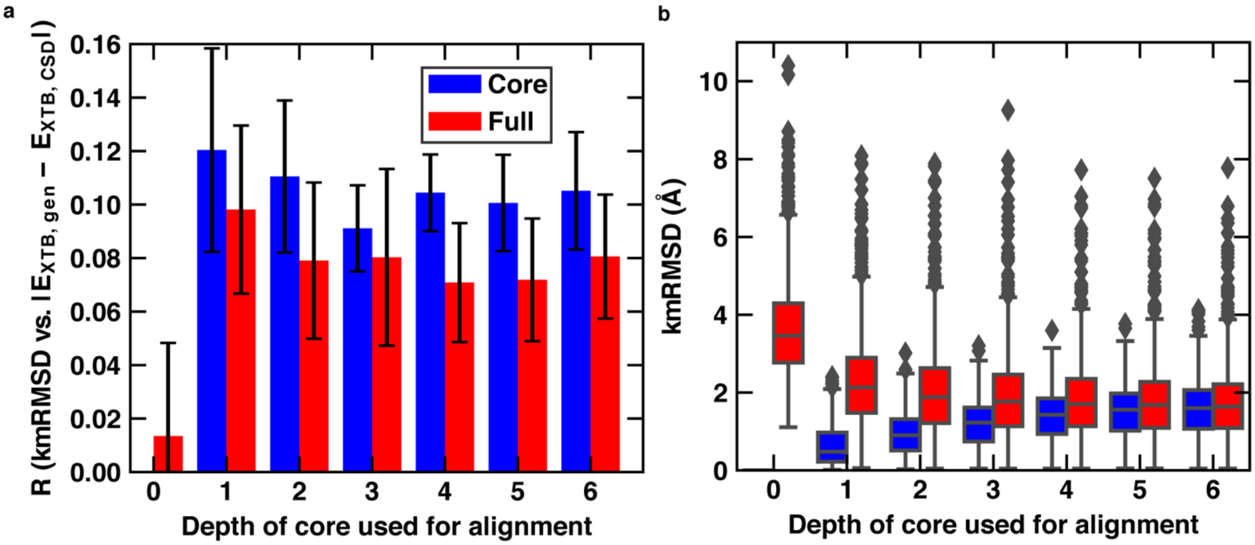

Supplementary Figure 11. a. Bar plots showing the Pearson R of the Kuhn–Munkres Root Mean Squared Distance (kmRMSD) alignment vs. absolute energy difference between the relaxed lowest-*xTB*-energy *Architector* complexes and the relaxed CSD structures. Bar heights are determined by the average Pearson R over 30 distinct randomly sampled subsets of 500 of 1,000 relaxed minimum-energy *Architector*-generated conformers over the CSD-replication dataset. Whiskers are from the standard deviation over the 30 randomly sampled subsets. **b.** boxplot of the kmRMSD values of both the core and full structures vs. depth of core used for alignment. Note that in a. the highest Pearson R including standard deviation confidence intervals is for core alignment using depth =1, but core alignment with depth=2 is nearly identical within the variance of the bars. At each point the core kmRMSD more strongly correlates with the absolute energy difference, indicating core kmRSMD without the pendent ligands captures more of the variation substantial to energy differences between mononuclear complexes. As depth of the core is increased, the core kmRMSD and full kmRMSD converge towards the same values due to more of the complexes being used to align the generated and CSD structures. In b. note that after core depth 0, the mean values of core kmRMSD increase with added depth, with core kmRMSD based on depth 2 showing comparable core kmRMSD to depth=1, while the full kmRMSD at depth 2 alignment falls slightly lower than at depth=1, indicating the depth=2 alignment improves ligand alignment over the simple depth 1. The full kmRMSD values tend to be higher overall due to the relative importance placed on ligand conformations that can more widely differ between similar structures further from the metal center.

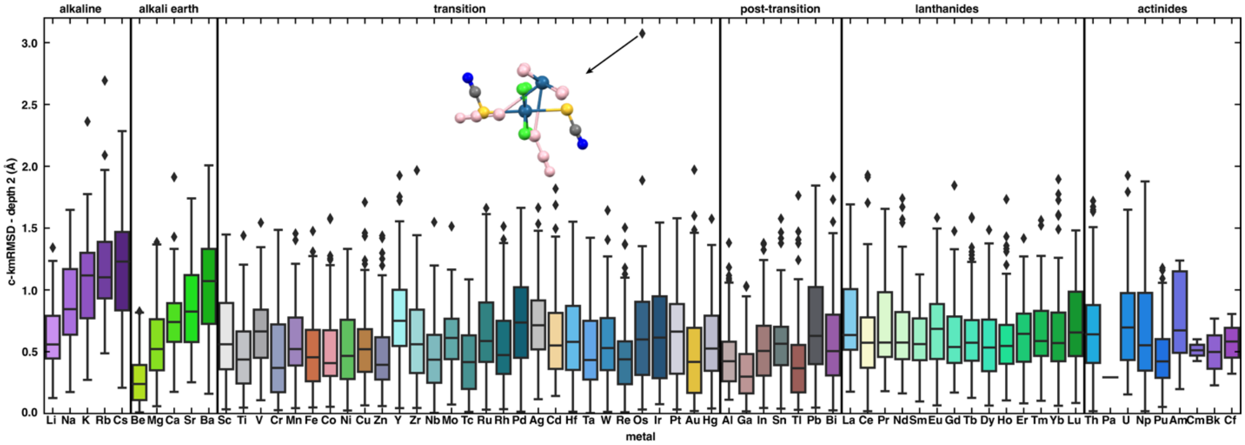

Supplementary Figure 12. Boxplot distributions of core (depth=2) kmRMSD (c-kmRMSD) of all minimum-c-kmRMSD *Architector*-generated complexes. All the boxplots are colored by jmol colors of each metal. Boxplots contain colored regions indicate from the 25 to the 75 percentile ranges name the inner quartile range (IQR), while whiskers indicate points within 1.5*(IQR) of either the 25 and 75 percentile points, and black diamonds indicate outliers beyond the 1.5*IQR cutoffs. Note that across the full periodic table the core depth 2 kmRMSD values average from 0.25-1 (Å) with exceptions in the alkaline metals falling higher than this region. Consistent with difficulties in generating the structures and larger ionic radii, alkaline metals show the largest average deviations from the CSD structures. The single outlier in transition metal and post transition metal space is highlighted as an inset corresponding to a structure where the trans configuration for the Thiocyanate ligands appear to not have converged in *xTB* resulting in larger c-kmRMSD.

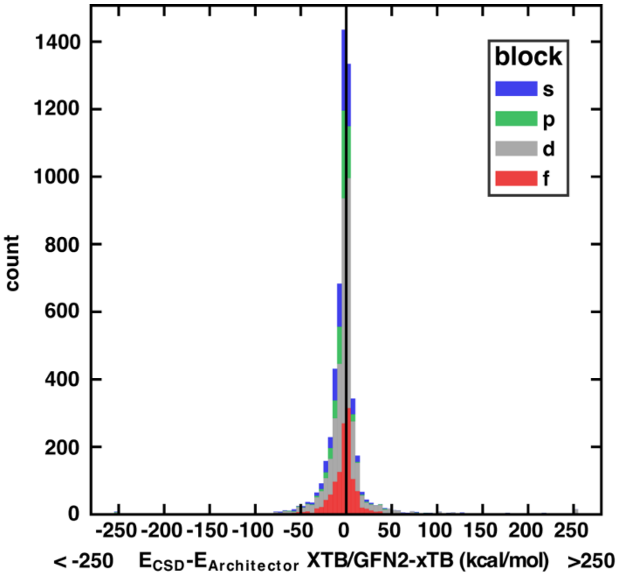

Supplementary Figure 13. Histogram of the difference in energies of the *xTB*/GFN2-xTB-relaxed CSD structure and the lowest-energy *Architector* conformers on the full CSD complex energetics. Note that trends with f-block element energetics (Figure 4) are closely followed by s-, p-, and d-block energetics.

Supplementary Note 11. ***CREST*** **implementation details.**

Crest sampling was performed with *CREST* version 2.12 with the following command line options:

crest structure.xyz --gfn2//gfnff --chrg CHRG --uhf UHF --notopo --quick > output.crest

Where the CHRG is the molecular charge of the molecule and UHF is the number of unpaired electrons in the molecule. This implementation performs only a single point calculation of GFN2-xTB at the structures relaxed at the GFN-FF level of theory (Upper Figure S14) so for comparison to Architector GFN2-xTB-relaxed conformers, a second relaxation is performed with the same relaxation methods utilized in Architector (Lower Figure S14).

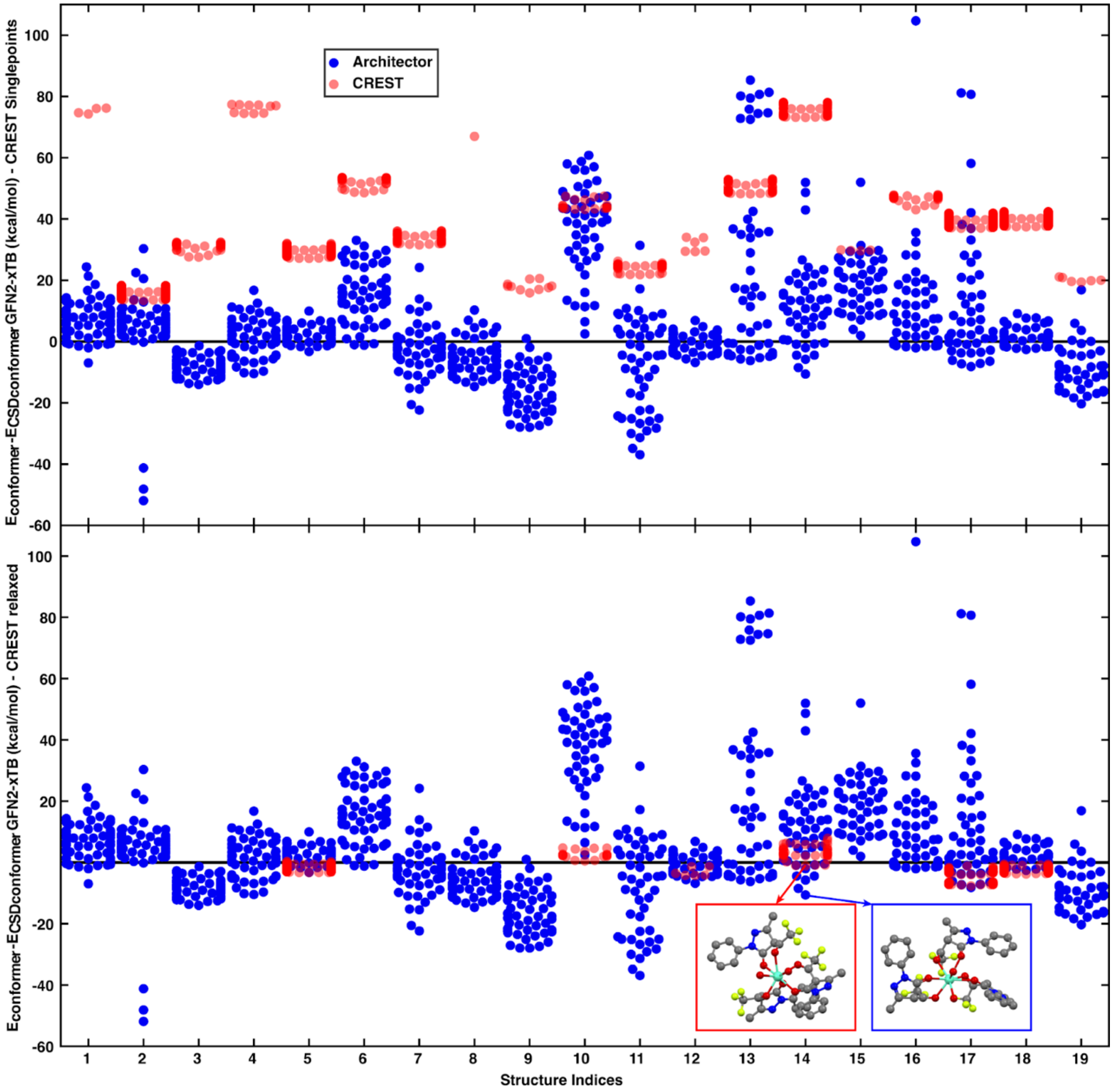

Supplementary Figure 14. *CREST* sampling vs *Architector* distributions in comparison to CSD structures evaluated with GFN2-xTB. *CREST* conformers were produced for 19 structures directly sampled from the CSD and were sampled with no final GFN2-xTB geometry relaxation (upper) and final GFN2-xTB geometry relaxation (lower). The geometry relaxation showed that the distributions are nearly as tight after relaxing in *CREST*, and align the energetics closer to the CSD and *Architector* structures. Several of the *CREST*-generated structures had difficulty converging during geometry relaxation and have been omitted. Note that *CREST* produces a tight distribution closer in energy to the original CSD structure while *Architector* produces more energetically diverse structures. Additionally, *Architector* will often generate structures at or lower in energy than the CSD structure with lowest-energy structures isoenergetic with *CREST*-generated conformers. In several cases, *Architector* structures are lower in energy than the lowest-*CREST* sampled structure due to sampling different relative symmetries of the ligands (see the insets highlighting the example from structure 14).

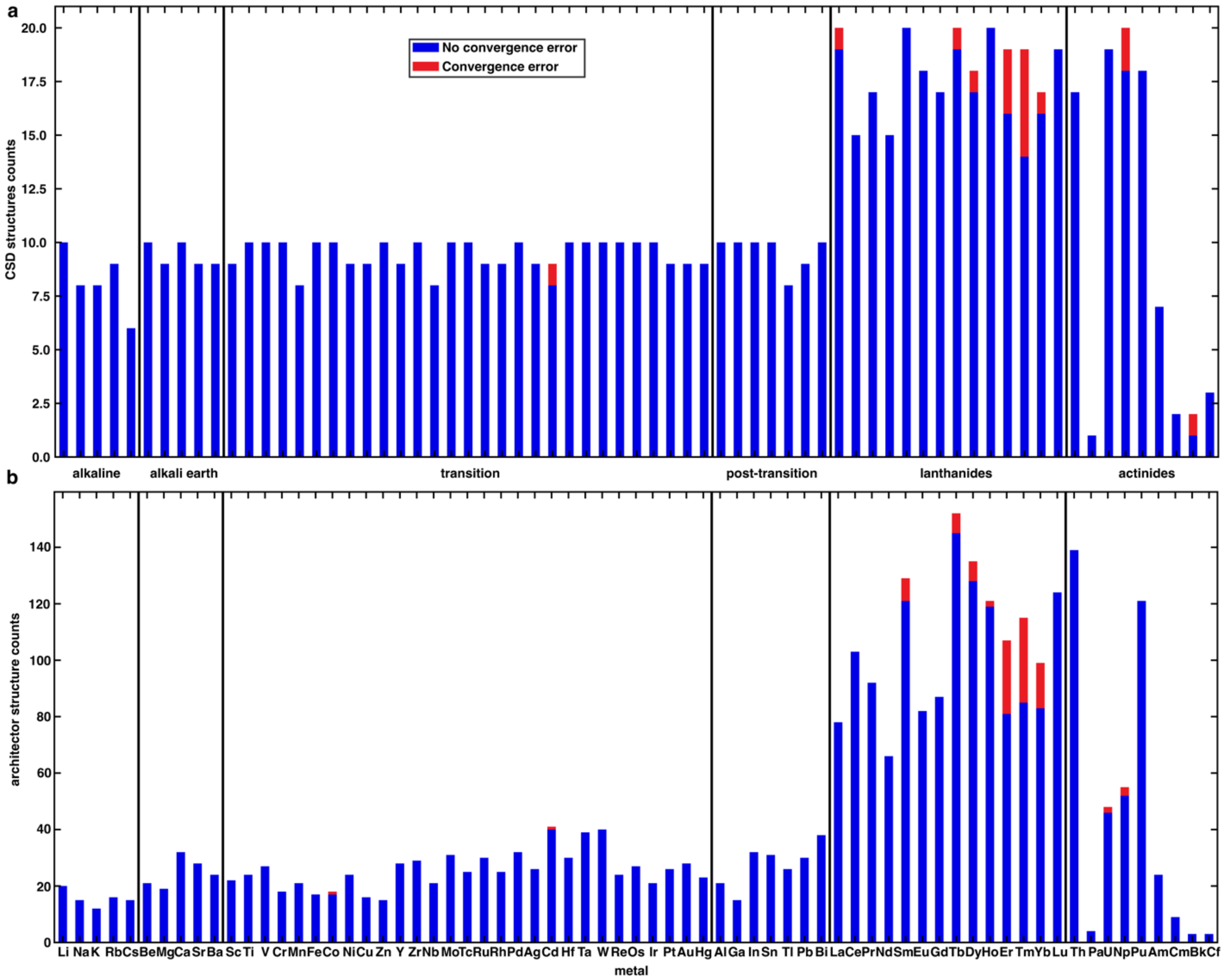

Supplementary Figure 15. a. DFT (PBE/DZP) self-consistent field (SCF) convergence rates directly on CSD-derived structures. **b.** Stacked barplots indicating SCF convergence of DFT calculations on *Architector*-generated structures. No convergence error indicates a calculation was able to run to completion without any warnings while convergence errors either failed to converge an SCF iteration. Note that more lanthanide and actinide complexes were tested to get better statistics and comparison for the f-block metals. Consistently, *Architector* structures are converging at similar if not better rates than CSD structures. The lower rates of convergence for lanthanides agrees with other work where converging the self-consistent field (SCF) for lanthanide complexes was particularly difficult.[21-23] When considering lanthanides in high-throughput electronic structure studies, additional care is suggested in selecting methods and SCF convergence parameters for desired properties. Such parameters were not pursued here for the purpose of general testing across the periodic table.

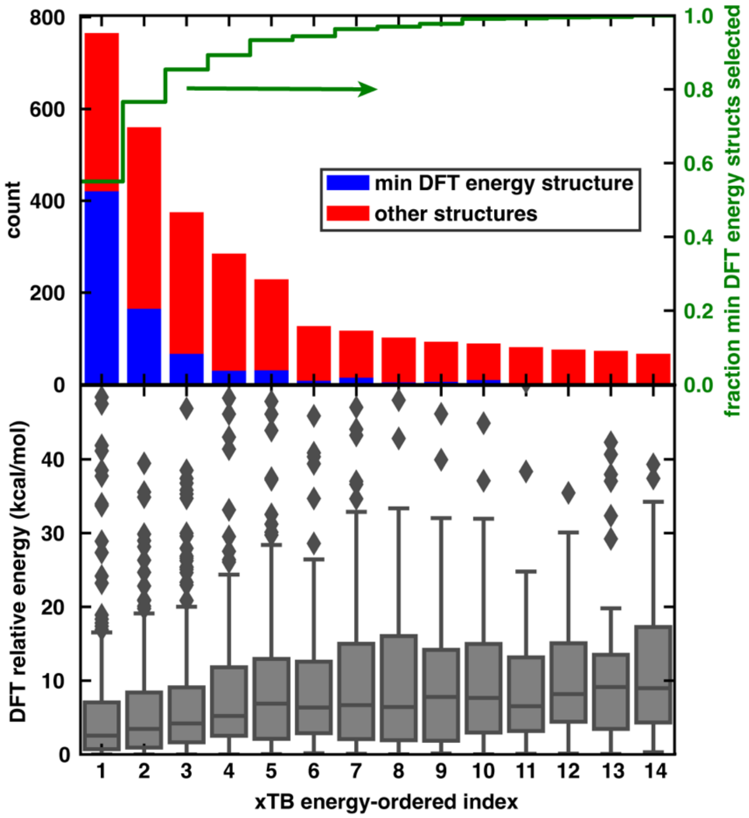

Supplementary Figure 16. *xTB* (GFN2-xTB) energy-ordered index versus both the fraction of minimum-energy DFT (PBE+D/DZP) energy structures (upper) and the DFT relative energy (E-Emin DFT) of the different conformers for conformers with DFT relative energy > 0 (lower). Note that a large fraction (93%) of lowest-energy DFT conformers is found within the first 5-lowest *xTB* conformers, and that generally the DFT relative energy increases from lower *xTB* indices to higher *xTB* indices.

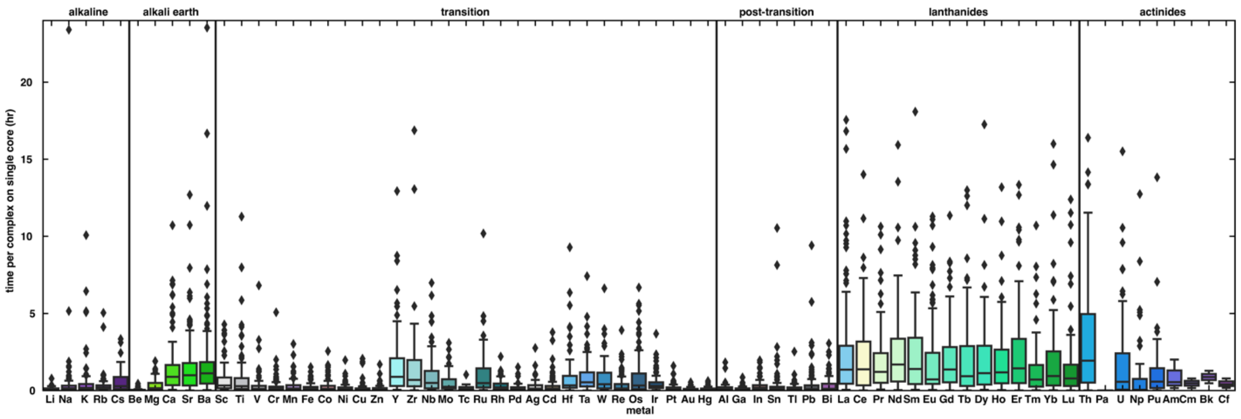

Supplementary Figure 17. Boxplot distributions of time per *Architector*-generated complexes. All the boxplots are are colored by jmol colors of each metal. Boxplots contain colored regions indicate from the 25 to the 75 percentile ranges name the inner quartile range (IQR), while whiskers indicate points within 1.5*(IQR) of either the 25 and 75 percentile points, and black diamonds indicate outliers beyond the 1.5*IQR cutoffs. Note that time for lanthanides and actinide complexes can be readily accelerated by GFN-FF assembly.

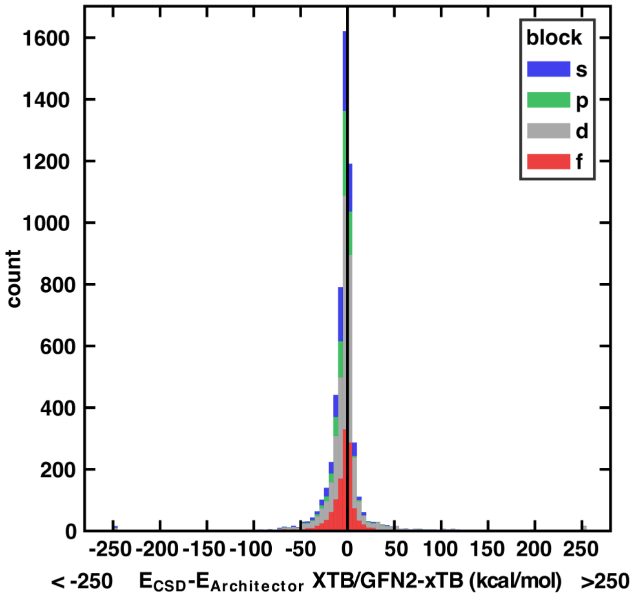

Supplementary Figure 18. Energetic comparison between the CSD structure and minimum-energy *Architector* structures evaluated with *xTB*/GFN2-xTB with Methanol implicit solvent added. Note that the distribution is quite similar and the mean of this plot is within 0.8 kcal/mol of the non-solvent applied GFN2-xTB method.

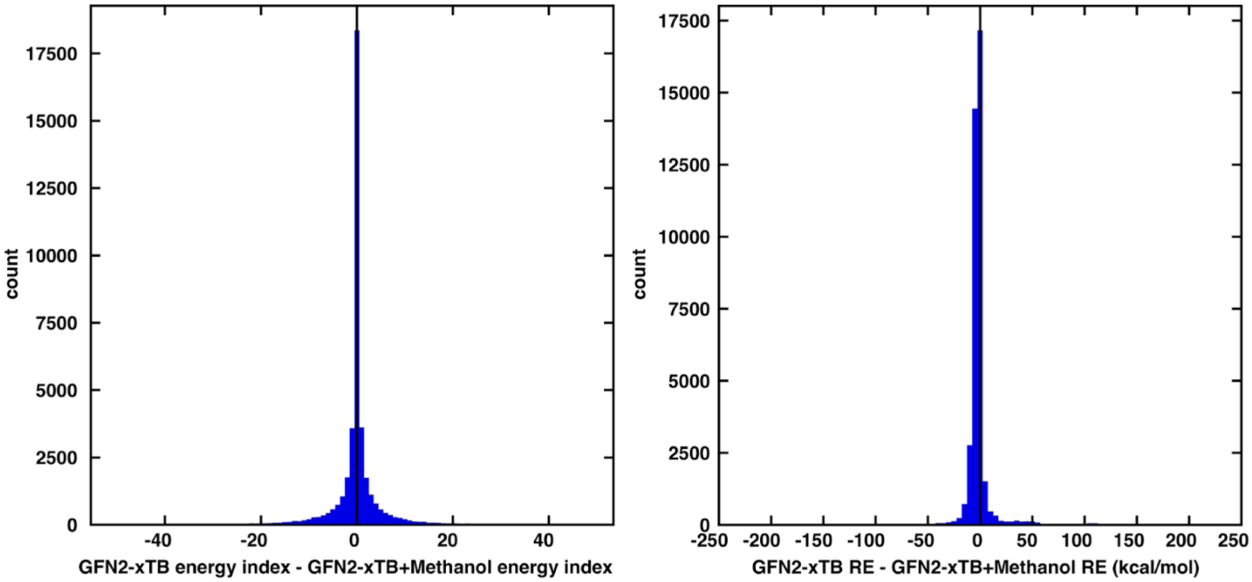

Supplementary Figure 19. *xTB* solvent effect impacts on conformer energetics. The difference between the energy-ordered index without solvent and with solvent across all conformers reported in the manuscript (left) with a mean deviation of 3 index orders between the two methods. The difference between relative energetics (RE, energy - lowest conformer energy) (right) with a mean deviation of 5 kcal/mol).

Supplementary Note 12. Single point comparisons were also performed to measure potential basis set and functional dependence of conformer rankings. PBE[24]+D[25,26]/DZP vs. PBE+D/TZ2P[27] calculations over several conformers revealed potential basis set superposition errors (BSSE) related to using smaller basis sets with energetic MAEs of 1.96 kcal/mol (Figure S20). B3LYP[28]+D/DZP and PBE+D/DZP calculations revealed relative conformer energetic MAEs between methods of 1.33 kcal/mol from different DFT functionals (Figure S20). Importantly, though basis set and functional choice affect energetics, we find they have relatively minor trends in energetics (Figure S20).

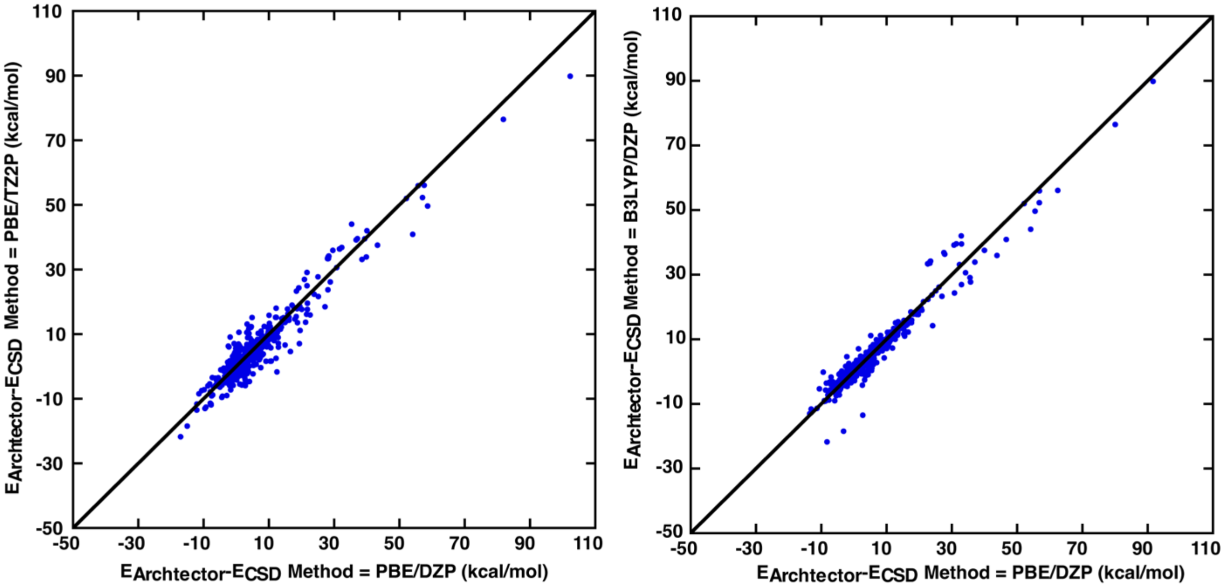

Supplementary Figure 20. Single point basis set (left) and method/functional (right) comparisons for DFT(+D) methods run on 850 PBE/DZP conformers from 100 structures with 42 different metal centers (primarily Lanthanides: 54%, Actinides: 27%, and d-Block metals: 13%) generated from *Architector*. Plots show parity between the base reported method PBE/DZP and larger basis set (TZ2P: left, MAE=1.96 kcal/mol) and different DFT functional (B3LYP/DZP: right, MAE=1.33 kcal/mol). Note that generally between different methods for functional and basis set, trends in energetics are maintained.

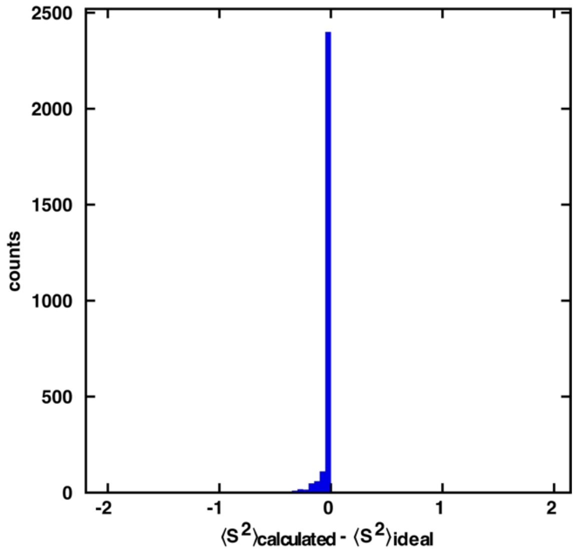

Supplementary Figure 21. Spin contamination plot. Note that 99.8% of structures do not contain spin contamination greater than 0.5. This indicates only a very small number of complexes generated by *Architector* result in spin contaminated electronic structure solutions.

Supplementary References:

1 Rego, N. & Koes, D. 3Dmol.js: molecular visualization with WebGL. *Bioinformatics* **31**, 1322-1324 (2015). https://doi.org:10.1093/bioinformatics/btu829

2 Kluyver, T. *et al.* in *20th International Conference on Electronic Publishing (01/01/16)* (eds Fernando Loizides & Birgit Scmidt) 87-90 (IOS Press, 2016).

3 Alvarez, S. *et al.* Shape maps and polyhedral interconversion paths in transition metal chemistry. *Coordination Chemistry Reviews* **249**, 1693-1708 (2005). https://doi.org:10.1016/j.ccr.2005.03.031

4 Groom, C. R., Bruno, I. J., Lightfoot, M. P. & Ward, S. C. The Cambridge Structural Database. *Acta Crystallographica Section B Structural Science, Crystal Engineering and Materials* **72**, 171-179 (2016). https://doi.org:10.1107/s2052520616003954

5 Pearson, K. LIII. On lines and planes of closest fit to systems of points in space. *The London, Edinburgh, and Dublin Philosophical Magazine and Journal of Science* **2**, 559-572 (1901). https://doi.org:10.1080/14786440109462720

6 Pyykkö, P. Additive Covalent Radii for Single-, Double-, and Triple-Bonded Molecules and Tetrahedrally Bonded Crystals: A Summary. *The Journal of Physical Chemistry A* **119**, 2326-2337 (2015). https://doi.org:10.1021/jp5065819

7 Alvarez, S. A cartography of the van der Waals territories. *Dalton Transactions* **42**, 8617 (2013). https://doi.org:10.1039/c3dt50599e

8 Blaney, J. M. & Dixon, J. S. in *Reviews in Computational Chemistry* 299-335 (1994).

9 Crippen, G. M. H. T. F. *Distance geometry and molecular conformation*. (Research Studies Press ; Wiley, 1988).

10 Rappe, A. K., Casewit, C. J., Colwell, K. S., Goddard, W. A. & Skiff, W. M. UFF, a full periodic table force field for molecular mechanics and molecular dynamics simulations.

Journal of the American Chemical Society **114**, 10024-10035 (1992). https://doi.org:10.1021/ja00051a040
11 Vogelstein, J. T. *et al.* Fast Approximate Quadratic Programming for Graph Matching. *PLOS ONE* **10**, e0121002 (2015). https://doi.org:10.1371/journal.pone.0121002
12 Virtanen, P. *et al.* SciPy 1.0: fundamental algorithms for scientific computing in Python. *Nature Methods* **17**, 261-272 (2020). https://doi.org:10.1038/s41592-019-0686-2
13 Halgren, T. A. Merck molecular force field. I. Basis, form, scope, parameterization, and performance of MMFF94. *Journal of Computational Chemistry* **17**, 490-519 (1996). https://doi.org:https://doi.org/10.1002/(SICI)1096-987X(199604)17:5/6<490::AID-JCC1>3.0.CO;2-P
14 Kabsch, W. A solution for the best rotation to relate two sets of vectors. *Acta Crystallographica Section A* **32**, 922-923 (1976). https://doi.org:10.1107/s0567739476001873
15 O'Boyle, N. M. *et al.* Open Babel: An open chemical toolbox. *Journal of Cheminformatics* **3**, 33 (2011). https://doi.org:10.1186/1758-2946-3-33
16 Bruno, I. J. *et al.* New software for searching the Cambridge Structural Database and visualizing crystal structures. *Acta Crystallographica Section B Structural Science* **58**, 389-397 (2002). https://doi.org:10.1107/s0108768102003324
17 Weininger, D. SMILES, a chemical language and information system. 1. Introduction to methodology and encoding rules. *Journal of Chemical Information and Modeling* **28**, 31-36 (1988). https://doi.org:10.1021/ci00057a005
18 McKay, B. D. & Piperno, A. Practical graph isomorphism, II. *Journal of Symbolic Computation* **60**, 94-112 (2014). https://doi.org:10.1016/j.jsc.2013.09.003
19 mendeleev - A Python resource for properties of chemical elements, ions and isotopes v. 0.12.1 (2014).
20 Taylor, M. G. *et al.* Seeing Is Believing: Experimental Spin States from Machine Learning Model Structure Predictions. *The Journal of Physical Chemistry A* **124**, 3286-3299 (2020). https://doi.org:10.1021/acs.jpca.0c01458
21 Calvello, S., Piccardo, M., Rao, S. V. & Soncini, A. CERES: An ab initio code dedicated to the calculation of the electronic structure and magnetic properties of lanthanide complexes. *Journal of Computational Chemistry* **39**, 328-337 (2018). https://doi.org:10.1002/jcc.25113
22 Martínez-Flores, C., Bolívar-Pineda, L. M. & Basiuk, V. A. Lanthanide bisphthalocyanine single-molecule magnets: A DFT survey of their geometries and electronic properties from lanthanum to lutetium. *Materials Chemistry and Physics* **287**, 126271 (2022). https://doi.org:https://doi.org/10.1016/j.matchemphys.2022.126271
23 Basiuk, V. A., Prezhdo, O. V. & Basiuk, E. V. Thermal smearing in DFT calculations: How small is really small? A case of La and Lu atoms adsorbed on graphene. *Materials Today Communications* **25**, 101595 (2020). https://doi.org:https://doi.org/10.1016/j.mtcomm.2020.101595
24 Perdew, J. P., Burke, K. & Ernzerhof, M. Generalized Gradient Approximation Made Simple. *Physical Review Letters* **77**, 3865-3868 (1996). https://doi.org:10.1103/physrevlett.77.3865
25 Caldeweyher, E. *et al.* A generally applicable atomic-charge dependent London dispersion correction. *The Journal of Chemical Physics* **150**, 154122 (2019). https://doi.org:10.1063/1.5090222

26 Grimme, S., Antony, J., Ehrlich, S. & Krieg, H. A consistent and accurate ab initio parametrization of density functional dispersion correction (DFT-D) for the 94 elements H-Pu. *The Journal of Chemical Physics* **132**, 154104 (2010). https://doi.org:10.1063/1.3382344

27 Van Lenthe, E. & Baerends, E. J. Optimized Slater-type basis sets for the elements 1-118. *Journal of Computational Chemistry* **24**, 1142-1156 (2003). https://doi.org:10.1002/jcc.10255

28 Stephens, P. J., Devlin, F. J., Chabalowski, C. F. & Frisch, M. J. Ab Initio Calculation of Vibrational Absorption and Circular Dichroism Spectra Using Density Functional Force Fields. *The Journal of Physical Chemistry* **98**, 11623-11627 (1994). https://doi.org:10.1021/j100096a001
